# Methods in causal inference. Part 1: causal diagrams and confounding

**DOI:** 10.1017/ehs.2024.35

**Published:** 2024-09-27

**Authors:** Joseph A. Bulbulia

**Affiliations:** Victoria University of Wellington, Wellington, New Zealand

**Keywords:** Causal inference, culture, DAGs, evolution, tutorial

## Abstract

Causal inference requires contrasting counterfactual states under specified interventions. Obtaining these contrasts from data depends on explicit assumptions and careful, multi-step workflows. Causal diagrams are crucial for clarifying the identifiability of counterfactual contrasts from data. Here, I explain how to use causal directed acyclic graphs (DAGs) to determine if and how causal effects can be identified from non-experimental observational data, offering practical reporting tips and suggestions to avoid common pitfalls.

## Graphical abstract

**Figure:**
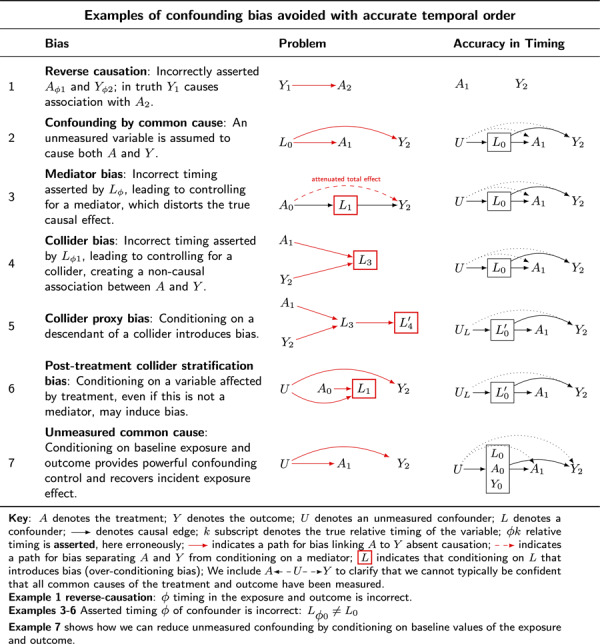

**Media summary:** Causal directed acyclic graphs (causal DAGs) are powerful tools for clarifying assumptions required for causal inference. However, they can be easily misused. This tutorial provides guidance on safely integrating causal diagrams into analytic workflows, underscoring the importance of starting with clearly defined causal questions.

## Introduction

Human research begins with two fundamental questions:

1. What do I want to know?
2. For which population does this knowledge generalise?

In the human sciences, our questions are typically causal. We aim to understand the effects of interventions on certain variables. However, many researchers collect data, apply complex regressions, and report model coefficients without understanding that the assumptions needed to support causal inferences differ from those needed to support predictions. Even when our models predict well, it typically remains unclear how these predictions relate to the scientific questions that sparked our interest.

Some say that association cannot imply causation and prohibit causal inferences from observational data. However, our experimental traditions reveal that when interventions are controlled and randomised, the coefficients we recover from statistical models can permit causal interpretations. The thread to causal inference is not from associations but rather from confounding. Despite familiarity with experimental protocols, however, many researchers struggle to address confounding by emulating randomisation and control using non-experimental or ‘real-world’ data. Practices of confounding control are not systematic. Indeed, we often overlook that what we take as control can inadvertently undermine our ability to consistently estimate causal effects, even in experiments (Montgomery et al., 2018). Although the term ‘crisis’ is arguably overused in the human sciences, the state of causal inference leaves considerable headroom for improvement. ‘Headroom for improvement’ applies to poor experimental designs that unintentionally weaken causal claims (Bulbulia, 2024d; Hernán et al., 2017; Montgomery et al., 2018).

Fortunately, recent decades have seen considerable progress in causal data science, commonly called ‘causal inference’, or ‘CI’. The progress has transformed those areas of health science, economics, political science and computer science that have adopted it. Causal inference provides methods for obtaining valid causal inferences from data through careful, systematic workflows. Within the workflows of causal inference, causal directed acyclic graphs (causal DAGs) – are powerful tools for evaluating whether and how causal effects can be identified from data. My purpose here is to explain where these tools fit within causal inference workflows and to illustrate several practical applications. I focus on causal directed acyclic graphs because they are relatively easy to use and clear for most applications. However, causal DAGs can be misused. Here, I consider common pitfalls and how to avoid them.

In Part 1, I review the conceptual foundations of causal inference. The basis of all causal inference lies in counterfactual contrasts. Although there are slightly different philosophical approaches to counterfactual reasoning, it is widely agreed that to infer a causal effect is to contrast counterfactuals for a well defined population under different levels of intervention. The overview I present here builds on the Neyman–Rubin potential outcomes framework of causal inference (Holland, 1986) as it has been extended for longitudinal treatments by epidemiologist James Robins (Robins, 1986).

In Part 2, I describe how causal DAGs allow investigators to evaluate whether and how causal effects may be identified from data using assumptions encoded in a causal DAG. I outline five elementary graphical structures from which all causal relations may be derived; these structures form the building blocks of every causal directed acyclic graphs. I then examine five rules that clarify whether and how investigators may identify causal effects from data under the structural (or equivalently causal) assumptions that a causal DAG encodes.

In Part 3, I apply causal directed acyclic graphs to seven common identification problems, showing how repeated-measures data collection addresses these problems. I then use causal diagrams to explain the limitations of repeated-measures data collection for identifying causal effects, tempering enthusiasm for easy solutions from repeated-measures designs.

In Part 4, I offer practical suggestions for creating and reporting causal directed acyclic graphs in scientific research. Where there is ambiguity or debate about how a treatment may be related to an outcome independently of causality, I suggest that investigators report multiple causal diagrams and conduct distinct analyses for each.

## Part 1: causal inference as counterfactual data science

The first step in every causal inference workflow is to state a well-defined causal question and a target population for whom answers are meant to generalise (Hernán et al., 2016a).

1. What causal quantity do I want to learn from the data?
2. For which population does this knowledge generalise?

Causal diagrams come after we have stated a causal question and have clarified our ‘target population’. Before reviewing causal diagrams we must consider what is required to answer these questions precisely.

### The fundamental problem of causal inference: missing counterfactual observations

We begin with the concept of causality itself. Consider an intervention, *A*, and its effect, *Y*. We say that *A* causes *Y* if altering *A* would lead to a change in *Y* (Hume, 1902; Lewis, 1973). If altering *A* would not change *Y*, we say that *A* has no causal effect on *Y*.

In causal inference, we aim to use data to quantitatively contrast the potential outcomes in response to different levels of a well-defined intervention. Commonly, we refer to such interventions as ‘exposures’ or ‘treatments’; we refer to the possible effects of interventions as ‘potential outcomes’.

Consider a binary treatment variable *A* ∈ {0, 1}. For each unit *i* in the set {1, 2, …, *n*}, when *A*_*i*_ is set to 0, the potential outcome under this condition is denoted *Y*_*i*_(0). Conversely, when *A*_*i*_ is set to 1, the potential outcome is denoted *Y*_*i*_(1). We refer to the terms *Y*_*i*_(1) and *Y*_*i*_(0) as ‘potential outcomes’ because, until realised, the effects of interventions describe counterfactual states.

Suppose that each unit *i* receives either *A*_*i*_ = 1 or *A*_*i*_ = 0. The corresponding outcomes are realised as *Y*_*i*_|*A*_*i*_ = 1 or *Y*_*i*_|*A*_*i*_ = 0. For now, we assume that each realised outcome under that intervention is equivalent to one of the potential outcomes required for a quantitative causal contrast, such that [(*Y*_*i*_(*a*)|*A*_*i*_ = *a*)] = (*Y*_*i*_|*A*_*i*_ = *a*). Thus, when *A*_*i*_ = 1, *Y*_*i*_(1)|*A*_*i*_ = 1 is observed. However, when *A*_*i*_ = 1, it follows that *Y*_*i*_(0)|*A*_*i*_ = 1 is not observed:Conversely:
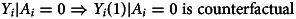
We define *δ*_*i*_ as the individual causal effect for unit *i* and express the individual causal effect as:
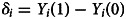
Notice that at the level of the individual, a causal effect is a contrast between treatments one of which is excluded by the other at any given time. That individual causal effects cannot be identified from observations is known as ‘*the fundamental problem of causal inference*’ (Holland, 1986; Rubin, 1976).

### Identifying causal effects using randomised experiments

Although it is not typically feasible to compute individual causal effects, under certain assumptions, it may be possible to estimate *average* treatment effects, also called ‘marginal effects’ by contrasting the outcomes of observed treatments among individuals who have been randomly assigned, perhaps conditional on measured covariates, to the treatment conditions that investigators wish to compare. We define an average treatment effect (ATE) as the difference between the expected or average outcomes observed under treatment where treatment has been randomly assigned, perhaps conditionally, on measured covariates. Consider a binary treatment,*A* ∈ {0, 1}. We write the average treatment effect as a contrast in the expected means of a population all of whose members are exposed to two levels of treatment:
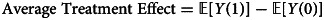

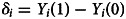
This is our pre-specified estimand for our target population. Note that a challenge remains in computing these treatment-group averages, given that individual causal effects are unobservable: each treatment to be compared is not administered to every member of the population from which a sample is drawn. We can frame the problem by referring to the *full data* required to compute this estimand – that is, in terms of the complete counterfactual dataset where the missing potential outcomes, inherent in observational data, were somehow available for everyone in the target population. Suppose that 50% of the sample is randomly assigned to each treatment condition. We find that for each treatment condition, half the observations over the joint distribution of the counterfactual data are inherently unobservable:
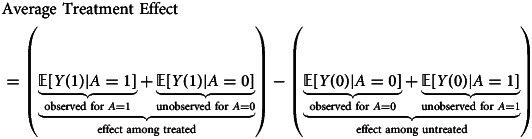
Although the fundamental problem of causal inference remains at the individual level, randomisation allows investigators to recover treatment group averages. When investigators randomise units into treatment conditions, ensuring full adherence and a sufficiently large sample to rule out chance differences in group composition, we can generally attribute differences in treatment group averages to the treatments themselves. That is, randomisation implies:
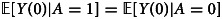
and
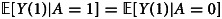

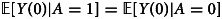
If we assume:
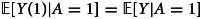
and
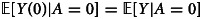
it follows that the average treatment effect of a randomised experiment can be computed:
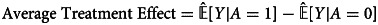
It is evident that we do not require the joint distribution over the full data (i.e. the counterfactual data) to obtain these averages. Rather, randomisation allows us to obtain a contrast of averages (or equivalently the average of contrasts) from the observed data.

Consider how randomised experiments enable average treatment effect estimation.

First, we must specify a population for whom we seek to generalise our results. We refer to this population as the *target population*. If the study population differs from the target population in the distribution of covariates that interact with the treatment, we will have no guarantees that our results will generalise (for discussions of sample/target population mismatch, refer to Imai et al. (2008), Westreich et al. (2017, 2019), Pearl and Bareinboim (2022), Bareinboim and Pearl (2013), Stuart et al. (2018) and Webster-Clark and Breskin (2021)).

Second, because the units in the study sample at randomisation may differ from the units in the study after randomisation, we must be careful to avoid biases that arise from sample/population mismatch over time (Bulbulia, 2024c; Hernán et al., 2004). If there is sample attrition or non-response, the treatment effect we obtain for the sample may differ from the treatment effect in the target population.

Third, a randomised experiment recovers the causal effect of random treatment assignment, not of the treatment itself. The effect of randomisation may differ from the effect of treatment if some participants do not adhere to the treatment to which they have been assigned (Hernán et al., 2017). The effect of randomised assignment is called the ‘intent-to-treat effect’ or equivalently the ‘intention-to-treat effect’. The effect of perfect adherence is called the ‘per-protocol effect’ (Hernán et al., 2017; Lash et al., 2020). To obtain the per-protocol effect for randomised experiments, methods for causal inference in observational settings must be applied (Bulbulia, 2024d; Hernán et al., 2017).

Fourth, I have presented the average treatment effect on the additive scale, that is, as an additive difference in average potential outcomes for the target population under two distinct levels of treatment. However, depending on the scientific question at hand, investigators may wish to estimate causal effects on the risk-ratio scale, the rate-ratio scale, the hazard-ratio scale or another scale. Where there are interactions such that treatment effects vary across different strata of the population, an estimate of the causal effect on the risk difference scale will differ in at least one stratum to be compared from the estimate on the risk ratio scale (Greenland, 2003; VanderWeele, 2012). The sensitivity of treatment effects in the presence of interactions to the scale of contrast underscores the importance of pre-specifying a scale for the causal contrast investigators hope to obtain.

Fifth, investigators may unintentionally spoil randomisation by adjusting for indicators that might be affected by the treatment, outcome or both, by excluding participants using attention checks, by collecting covariate data that might be affected by the experimental conditions, by failing to account for non-response and loss-to-follow-up, and by committing any number of other self-inflicted injuries (Bulbulia, 2024d). Unfortunately, such practices of self-inflicted injury are widespread (Montgomery et al., 2018). Notably, causal directed acyclic graphs are useful for describing risks to valid causal identification in experiments (refer to Hernán et al., 2017), a topic that I consider elsewhere (Bulbulia, 2024d).

In observational studies, investigators might wish to describe the target population of interest as a restriction of the study sample population. For example, investigators might wish to estimate the average treatment effect only in the population that received the treatment (Greifer et al., 2023; Greifer, 2023). This treatment effect is sometimes called the average treatment effect in the treated (ATT) and may be expressed as:
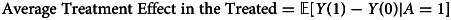
Consider that if investigators are interested in the average treatment effect in the treated, counterfactual comparisons are deliberately restricted to the sample population that was treated. Here, investigators seek to obtain the average of the missing counterfactual outcomes for the treated population had they not been treated, without also obtaining the missing counterfactual outcomes for the untreated population had they been treated. Identifying causal effects in a restricted population may imply different causal assumptions and analytic workflows. Supplementary materials S2 describe an example for which the assumptions required to estimate the average treatment effect in the treated might be preferred. Here, we use the term ATE as a placeholder to mean the average treatment effect, or equivalently the ‘marginal effect’, for a target population on a pre-specified scale of causal contrast, where we assume that this effect estimate pertains to the source population from which the analytic sample was randomly drawn (under the assumption of random sampling, refer to Dahabreh et al., 2019; Dahabreh & Hernán, 2019).

Setting aside the important detail that the ‘average treatment effect’ requires considerable care in its specification, it is worth pausing to marvel at how an ideally conducted randomised controlled experiment provides a means for identifying inherently unobservable counterfactuals. It does so by using a Sherlock Holmes method of inference by elimination of confounders, which randomisation balances across treatments.

When experimenters observe a difference in average treatment effects, and all else goes right, they may infer that the distribution of potential outcomes differs by treatment because randomisation exhausts every other explanation. Again, if the experiment is conducted properly, experimenters are entitled to this inference because randomisation balances the distribution of potential confounders across the treatment groups to be compared.

However, when treatment assignments have not been randomised, we typically lack guarantees that the variables that bias causal associations are balanced across treatment conditions. Unfortunately, randomised experiments are impractical for addressing many scientifically important questions. This bitter constraint is familiar to evolutionary human scientists. We often confront ‘What if?’ questions that are rooted in the unidirectional nature of human history. However, understanding how randomisation obtains the missing counterfactual outcomes that we require to consistently estimate average treatment effects clarifies the tasks of causal inference in non-experimental settings (Hernán et al., 2008a, 2022; Hernán & Robins, 2006a): we want to ensure balance in the variables that might affect outcomes under treatment in the treatment groups to be compared.

Next, we examine basic causal identification assumptions in greater detail. We do so because using causal diagrams without understanding these assumptions may lead to unwarranted false confidence.

### Fundamental assumptions required for causal inference in the potential outcomes framework

Three fundamental identification assumptions must be satisfied to consistently estimate causal effects from data. These assumptions are typically satisfied in properly executed randomised controlled trials but not in real-world studies where randomised treatment assignment is absent.

#### Assumption 1: causal consistency

We satisfy the causal consistency assumption when, for each unit *i* in the set {1, 2, …, *n*}, the observed outcome corresponds to one of the specific counterfactual outcomes to be compared such that:
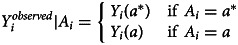
The causal consistency assumption implies that the observed outcome at the specific treatment level that an individual receives equates to that individual's counterfactual outcome at the observed treatment level. Although this assumption would appear straightforward, outside ideally controlled randomised experiments, treatment conditions typically vary, and treatment heterogeneity poses considerable challenges to satisfying this assumption. Refer to supplementary materials S3 for further discussion of how investigators may satisfy the causal consistency assumption in real-world settings.

#### Assumption 2: positivity

We satisfy the positivity assumption if there is a non-zero probability of receiving each treatment level within each stratum of covariate required to ensure conditional exchangeability of treatments (assumption 3). Where *A* is the treatment and *L* is a vector of covariates sufficient to ensure no unmeasured confounding, we say that positivity is achieved if:
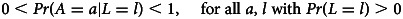
There are two types of positivity violation:

1. *Random non-positivity* – when a treatment is theoretically possible but specific treatment levels are not represented in the data, random non-positivity is the only identifiability assumption verifiable with data.
2. *Deterministic non-positivity* – when the treatment is implausible by nature, such as a hysterectomy in biological males.

Satisfying the positivity assumption can present considerable data challenges (Bulbulia et al., 2023; Westreich & Cole, 2010). For instance, if we wanted to estimate a one-year causal effect of weekly religious service attendance on charitable donations, controlling for baseline attendance, and the natural transition rate to weekly service attendance is low, the effective sample size for the treatment condition may be insufficient. Where the positivity assumption is violated, causal diagrams will be of limited utility because observations will not support valid causal inferences even in the absence of confounding biases. Supplementary materials S2 presents a worked example illustrating this difficulty in a cultural evolutionary context.)

#### Assumption 3: conditional exchangeability (also ‘no unmeasured confounding’, ‘conditional ignorability’, ‘d-separation’)

We satisfy the conditional exchangeability assumption if the treatment groups are conditionally balanced in the variables that could affect the potential outcomes. In experimental designs, random assignment facilitates this assumption. In observational studies effort is required to control for any covariate that could account for observed correlations between *A* and *Y* without a causal effect of *A* on *Y*.

Let 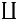 denote independence, and let *L* denote the set of covariates necessary to ensure this conditional independence. Conditional exchangeability is satisfied when:
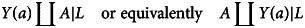
If we assume that the positivity and consistency assumptions also hold, we may compute the ATE on the difference scale:
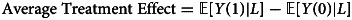
In randomised controlled experiments, exchangeability is unconditional. We would only adjust our statistical model by interacting the treatment with pre-treatment variables to improve efficiency (Lin, 2013) or diminish threats to valid randomisation from chance imbalances (Hernán & Robins, 2024). However, it would be confusing to think of such an adjustment as ‘control’.

In real-world observational studies, where measured covariates are sufficient to ensure conditional exchangeability across the treatment groups to be compared – also called, ‘no unmeasured confounding’ or ‘ignorability’ – we may obtain valid estimates for an average treatment effect by conditioning on the densities of measured confounders by treatment group. Where *A* = *a* and *A* = a* are the treatment levels we seek to contrast:By causal consistency, we obtain:
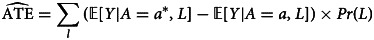
For continuous covariates *L*, we have:
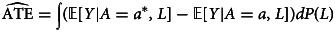
We may now state the primary function of a causal DAG, which is to identify sources of bias that may lead to an association between an exposure and outcome in the absence of causation. Causal DAGs visually encode features of a causal order necessary to evaluate the assumptions of conditional exchangeability, or equivalently of ‘no-unmeasured confounding’, or equivalently of ‘ignorability’ – or equivalently of ‘d-separation’ (explained next). Although causal directed acyclic graphs may be useful for addressing other biases such as measurement error and target-population restriction bias (also called ‘selection bias’) (Bulbulia, 2024c; Hernán & Robins, 2024), it is important to understand that causal directed acyclic graphs are specifically designed to evaluate the assumptions of conditional exchangeability or ‘d-separation’; any other use is strictly ‘off-label’.

Finally, it is important to emphasise that without randomisation, we typically cannot ensure that there is no-unmeasured confounding (Greifer et al., 2023; Stuart et al., 2015). For this reason, causal data science workflows typically include sensitivity analyses to determine how much unmeasured confounding would be required to compromise a study's findings (VanderWeele & Ding, 2017). Moreover, even if investigators do not represent unmeasured common causes of treatment and exposure in the causal DAGs they craft for observational studies, we should assume there are umeasured common causes and plan sensitivity analyses.

### Summary of Part 1

Causal data science is distinct from ordinary data science. The initial step involves formulating a precise causal question that clearly defines a treatment or sequence of treatments, the outcome or outcomes to be contrasted under treatment, and a population of interest called the target population. We must then satisfy the three fundamental assumptions required for causal inference, assumptions that are implicit in the ideal of a randomised controlled experiment: *causal consistency* – outcomes at the treatment levels to be compared must align with their counterfactual counterparts; *positivity* – each treatment must have a non-zero probability across all covariates; and *conditional exchangeability* – there should be no unmeasured confounding, meaning treatment assignment is ignorable conditional on measured confounders, or equivalently, that treatment groups are conditionally exchangeable.

## Part 2: how causal directed acyclic graphs clarify the conditional exchangeability assumption

Next, I will introduce causal DAGs. I will start by explaining the meaning of the symbols used. Table 1 summarises our terminology and conventions. Refer to supplementary materials S1 for a glossary of common causal inference terms.

**Table 1.** Variable naming conventions

Symbol	Description
*X*	A capital letter representing a random variable
*X* = *x*	A small letter indicating the random variable *X* fixed at value *x*
*A*	The treatment or, equivalently, the exposure
*A* = *a*	Treatment *A* fixed to level *a*
*Y*	The outcome variable
*Y*(*a*)	The potential or counterfactual outcome when *A* = *a*. Also represented as *Ya* or *Ya*
*L*	Measured confounder(s): typically comprises a set of variables
*U*	Unmeasured confounder
F	Effect-modifier (or ‘moderator’) of *A* on *Y*
*M*	Mediator of *A* on *Y*
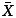	Sequential variables, e.g. ; 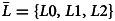
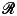	Denotes random treatment assignment
	A causal graph, here, a causal directed acyclic graph (DAG)

### Variable naming conventions

- *X* denotes a random variable without reference to its role.
- *A* denotes the ‘treatment’ or ‘exposure’ – a random variable. This is the variable for which we seek to understand the effect of intervening on it. It is the ‘cause’.
- *A* = *a* denotes a fixed ‘treatment’ or ‘exposure’. The random variable *A* is set to level *A* = *a*.
- *Y* denotes the outcome or response of an intervention. It is the ‘effect’.
- *Y*(*a*) denotes the counterfactual or potential state of *Y* in response to setting the level of the treatment to a specific level, *A* = *a*. The outcome *Y* is as would be observed when, perhaps contrary to fact, treatment *A* is set to level *A* = *a*. Different conventions exist for expressing a potential or counterfactual outcome, such as *Y*^*a*^,*Y*_*a*_.
- *L* denotes a measured confounder or set of confounders. This set, if conditioned upon, ensures that any differences between the potential outcomes under different levels of the treatment are the result of the treatment and not the result of a common cause of the treatment and the outcome. Mathematically, we write this independence:
- *U* denotes an unmeasured confounder or confounders. *U* is a variable or set of variables that may affect both the treatment and the outcome, leading to an association in the absence of causality, even after conditioning on measured covariates:
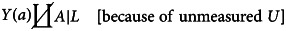
- *F* denotes a modifier of the treatment effect. *F* alters the magnitude or direction of the effect of treatment *A* on an outcome *Y*.
- *M* denotes a mediator, a variable that transmits the effect of treatment *A* on an outcome *Y*.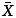 denotes a sequence of variables, for example, a sequence of treatments.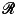 denotes a randomisation to treatment condition.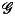 denotes a graph, here, a causal directed acyclic graph.

Note that investigators use a variety of different symbols. There is no unique right way to create a causal directed acyclic graph, except that the meaning must be clear and the graph must be capable of identifying relationships of conditional and unconditional independence between the treatment and outcome. Although directed acyclic graphs are accessible tools, general graphical models such as ‘Single World Intervention Graphs’, which allow for the explicit representation of counterfactual dependencies, may be preferable for investigators to estimate causal effects under multiple interventions (Bulbulia, 2024b; Richardson & Robins, 2013a).

### Conventions we use in this article to create causal directed acyclic graphs

The conventions we use to describe components of our causal graphs are given in Table 2.

- *Node* – a node or vertex represents characteristics or features of units within a population on a causal diagram – that is a ‘variable’. In causal directed acyclic graphs, we draw nodes with respect to the *target population*, which is the population for whom investigators seek causal inferences (Suzuki et al., 2020). Time-indexed node *X*_*t*_ denotes relative chronology; *X*_*ϕt*_ is our convention for indicating that timing is assumed, perhaps erroneously.
- *Edge without an arrow* 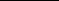 – path of association, causality not asserted.
- *Red edge without an arrow* (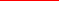) – confounding path, ignores arrows to clarify statistical dependencies.
- *Arrow* (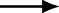) – denotes causal relationship from the node at the base of the arrow (a parent) to the node at the tip of the arrow (a child). We typically refrain from drawing an arrow from treatment to outcome to avoid asserting a causal path from *A* to *Y* because the function of a causal directed acyclic graph is to evaluate whether causality can be identified for this path.
- *Red arrow* (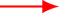) – path of non-causal association between the treatment and outcome.
- *Dashed arrow* (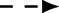) – denotes a true association between the treatment and outcome that becomes partially obscured when conditioning on a mediator, assuming *A* causes *Y*.
- *Dashed red arrow* (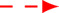) – highlights over-conditioning bias from conditioning on a mediator.
- *Open blue arrow* (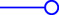) – highlights effect modification, occurring when the treatment effect levels vary within covariate levels. We do not assess the causal effect of the effect 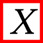 modifier on the outcome, recognising that intervening on the effect modifier may be incoherent. This is an off-label convention we use to clarify our interest in effect modification within strata of a covariate when there is a true treatment effect. However, it is possible to replace these open blue arrows with ordinary nodes and explain that the edges are drawn not for identification but for evaluating generalisations (see Bulbulia, 2024b).
- *Boxed variable* 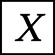 – conditioning or adjustment for *X*.
- *Red-boxed variable* 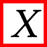 – highlights the source of confounding bias from adjustment.
- *Dashed circle* 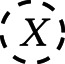 – no adjustment is made for a variable (implied for unmeasured confounders.)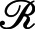 randomisation, for example, randomisation into treatment: 
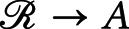
.
- *Presenting temporal order* – causal directed acyclic graphs must be – as truth in advertising implies – *acyclic.* Directed edges or arrows define ancestral relations. No descendant node can cause an ancestor node. Therefore causal diagrams are, by default, sequentially ordered.

**Table 2.** Nodes, edges, conditioning conventions

Symbol	Meaning	Example
*X*	*Node or vertex* – variable denoted by a letter	*A* (treatment), *Y* (outcome)
*Xt*	*Time-indexed node* – denotes relative chronology	*A*_1_ *Y*_0_
*Xϕt*	*Timing assumed but not known –* relative chronology asserted	*A*_*ϕ*1_ *Y*_*ϕ*2_
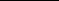	*Edge with no arrow –* association	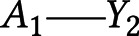
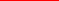	*Red association path –* confounding path: ignores arrows to clarify statistical dependencies	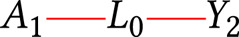
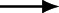	*Edge with an arrow –* here, denotes causal association	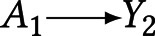
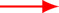	*Red arrow –* path through which bias flows	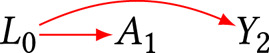
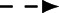	*Dashed arrow –* causal effect not through a mediator (direct effect)	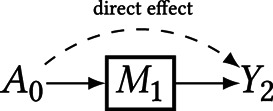
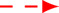	*Dashed red arrow –* biased total effect when conditioning on a mediator	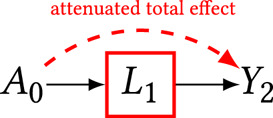
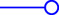	*Effect modification path –* assumes 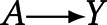 and focuses on the modification within levels of another variable. *Blue path is not evaluated for causality and need not have a causal interpretation*	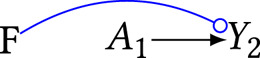
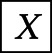	*Boxed variable –* conditioning/adjustment	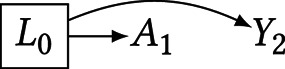
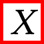	*Red boxed variable –* variable that when conditioned upon induces bias	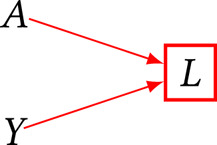
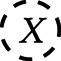	*Dashed circle –* no adjustment for variable	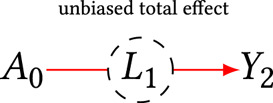
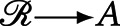	*Random treatment assignment –* such that 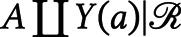	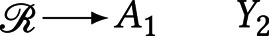

Nevertheless, to make our causal graphs more readable, we adopt the following conventions:

1. The layout of a causal diagram is structured from left to right to reflect the assumed sequence of causality as it unfolds.
2. We often index our nodes using *X*_*t*_ to indicate their relative timing and chronological order, where *t* represents the time point or sequence in the timeline of events.
3. Where temporal order is uncertain or unknown, we use the notation *X*_*ϕt*_ to propose a temporal order that is uncertain.

Typically, the timing of unmeasured confounders is unknown, except that they occur before the treatments of interest; hence, we place confounders to the left of the treatments and outcomes they are assumed to affect, but without any time indexing.

Again, temporal order is implied by the relationship of nodes and edges. However, explicitly representing the order in the layout of one's causal graph often makes it easier to evaluate, and the convention representing uncertainty is useful, particularly when the data do not ensure the relative timing of the occurrence of the variable in a causal graph.

More generally, investigators use various conventions to convey causal structures on graphs. Whichever convention we adopt must be clear.

Finally, note that all nodes and paths on causal graphs – including the absence of nodes and paths – are asserted. Constructing causal diagrams requires expert judgment of the scientific system under investigation. It is a great power given to those who construct causal graphs, and *with great power comes great responsibility to be transparent.* When investigators are unclear or there is debate about which graphical model fits reality, they should present multiple causal graphs. Where identification is possible in several candidate causal graphs, they should perform and report multiple analyses.

### How causal directed acyclic graphs relate observations to counterfactual interventions

#### Ancestral relations in directed acyclic graphs

We define the relation of ‘parent’ and ‘child’ on a directed acyclic graph as follows:

1. Node *A* is a *parent* of node *B* if there is a directed edge from *A* to *B*, denoted *A* → *B*.
2. Node *B* is a *child* of node *A* if there is a directed edge from *A* to *B*, denoted *A* → *B*.

It follows that a parent and child are *adjacent nodes* connected by a directed edge.

We denote the set of all parents of a node *B* as pa(*B*).

In a directed acyclic graph, the directed edge *A* → *B* indicates a statistical dependency where *A* may provide information about *B*. In a causal directed acyclic graph, the directed edge *A* → *B* is interpreted as a causal relationship, meaning *A* is a direct cause of *B*.

We further define the relations of *ancestor* and *descendant* on a directed acyclic graph as follows:

1. Node *A* is an *ancestor* of node *C* if there exists a directed path from *A* to *C*. Formally, *A* is an ancestor of *C* if there exists a sequence of adjacent nodes (*A*, *B*_1_, *B*_2_, …, *B*_*t*_, *C*) such that *A* → *B*_1_ → *B*_2_ → ⋅ ⋅ ⋅ → *B*_*t*_ → *C*.
2. Node *C* is a *descendant* of node *A* if there exists a directed path from *A* to *C*. Formally, *C* is a descendant of *A* if there exists a sequence of adjacent nodes (*A*, *B*_1_, *B*_2_, …, *B*_*t*_, *C*) such that *A* → *B*_1_ → *B*_2_ → ⋅ ⋅ ⋅ → *B*_*t*_ → *C*.

It follows that a node can have multiple ancestors and multiple descendants.

#### Markov factorisation and the local Markov assumption

Pearl (2009: 52) asks us to imagine the following. Suppose we have a distribution *P* defined on n discrete variables,*X*_1_, *X*_2_, …, *X*_*n*_. By the chain rule, the joint distribution for variables *X*_1_, *X*_2_, …, *X*_*n*_ on a graph can be decomposed into the product of *n* conditional distributions such that we may obtain the following factorisation:
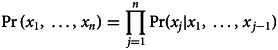
We translate nodes and edges on a graph into a set of conditional independences that a graph implies over statistical distributions.

According to *the local Markov assumption*, given its parents in a directed acyclic graph, a node is said to be independent of all its non-descendants. Under this assumption, we obtain what Pearl calls Bayesian network factorisation, such that:
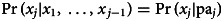
This factorisation greatly simplifies the calculation of joint distributions encoded in a directed acyclic graph (whether causal or non-causal) by reducing the complex factorisation of conditional distributions in 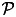 to simpler conditional distributions involving the parent set PA_*j*_, as represented by the structural components of the graph (Lauritzen et al., 1990; Pearl, 1988, 1995, 2009).

#### Minimality assumption

The minimality assumption combines (a) the local Markov assumption with (b) the assumption that adjacent nodes on the graph are dependent. The minimality assumption asserts that the DAG is minimal with respect to the set of conditional independencies it encodes. This means no edges can be removed from the graph without altering the set of implied conditional independencies. It ensures that all adjacent nodes are dependent, and the graph does not include any unnecessary edges (Neal, 2020).

#### Causal edge assumption

The causal edges assumption states that every parent is a direct cause of their children. Given the minimality assumption, the causal edges assumption allows us to interpret the conditional dependence between variables on a graph based on the causal relationships encoded by the arrangement of nodes and edges (Neal, 2020).

#### Compatibility assumption

The compatibility assumption ensures that the joint distribution of variables aligns with the conditional independencies implied by the causal graph. This assumption requires that the probabilistic model conforms to the graph's structural assumptions. Demonstrating compatibility directly from data is challenging, as it involves verifying that all conditional independencies specified by the causal DAG are present in the data. Therefore, we typically assume compatibility rather than attempt to empirically prove it (Pearl, 2009).

#### Faithfulness

A causal diagram is considered faithful to a given set of data if all the conditional independencies present in the data are accurately depicted in the graph. Conversely, the graph is faithful if every dependency implied by the graph's structure can be observed in the data (Hernán & Robins, 2024). Faithfulness ensures that the graphical representation of relationships between variables accords with empirical evidence (Pearl, 2009).

We may distinguish between *weak faithfulness* and *strong faithfulness*:

- *Weak faithfulness* allows for the possibility that some observed independencies might occur because specific parameter values cause cancellations. It acknowledges that some conditional independencies in the data may not be reflected in the graph’s structure because they result from exact numerical coincidences.
- *Strong faithfulness* requires that all and only the conditional independencies that hold in the data are exactly those implied by the graph via its d-separation properties. It rules out the possibility of independencies arising from exact cancellations in the parameters.

The faithfulness assumption, whether weak or strong, is not directly testable from observed data (Pearl, 2009).

#### d-Separation

In a causal diagram, a path is ‘blocked’ or ‘d-separated’ if a node along it interrupts causation. Two variables are d-separated if all paths connecting them are blocked, making them conditionally independent. Conversely, unblocked paths result in ‘d-connected’ variables, implying potential dependence (Pearl, 1995, 2009). (Note that ‘d’ stands for ‘directional’, emphasising that the separation considers the directionality of edges. This is crucial because the concept relies on the direction of the arrows in the DAG to determine independence.)

The rules of d-separation are as follows:

1. *Fork rule* 
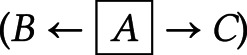
 –*B* and *C* are independent when conditioning on *A* (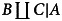).
2. *Chain rule* 
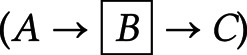
 – conditioning on *B* blocks the path between *A* and *C* (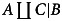).
3. *Collider rule* 
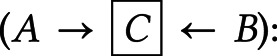
 –*A* and *B* are marginally independent. However, conditioning on *C* or any of its descendants introduces a dependence between *A* and *B* such that (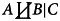).

Judea Pearl proved d-separation in the 1990s (Pearl, 1995, 2009).

It follows from d-separation that:

1. An open path (no variables conditioned on) is blocked only if two arrows point to the same node: *A* → *C* ← *B*. The node of common effect (here *C*) is called a *collider*.
2. Conditioning on a collider does not block a path; thus, 
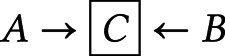
 can lead to an association between *A* and *B* in the absence of causation.
3. Conditioning on a descendant of a collider opens a path; for example if 
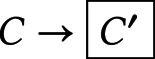
, then 
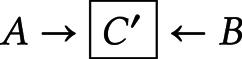
 is open.
4. If a path does not contain a collider, any variable conditioned along the path blocks it; thus, 
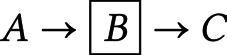
 blocks the path from *A* to *C* (Hernán & Robins, 2024: 78; Pearl, 2009). Thus, in paths without colliders, conditioning on any node along the path blocks the path. In paths with colliders, conditioning on the collider or its descendants unblocks the path.

#### Backdoor adjustment

From d-separation, Pearl was able to define a general identification algorithm for causal identification, called the ‘backdoor adjustment theorem’ (Pearl, 2009).

Let us shift to the general notation that we will use in the following examples. Where *A* denotes the treatment, *Y* denotes the outcome and *L* denotes a set (or subset) of measured covariates. In a causal directed acyclic graph (causal DAG), we say that a set of variables *L* satisfies the backdoor adjustment theorem relative to the treatment *A* and the outcome *Y* if *L* blocks every path between *A* and *Y* that contains an arrow pointing into *A* (a backdoor path). Formally, *L* must satisfy two conditions:

1. No element of *L* is a descendant of *A*.
2. *L* blocks all backdoor paths from *A* to *Y* (there are no unmeasured confounders affecting both *A* and *Y* other than *L*.)

If *L* satisfies these conditions, the causal effect of *A* on *Y* can be estimated by conditioning on 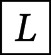 (Pearl, 2009).

#### Front door path criterion

Pearl also proves a ‘front-door adjustment’ criterion, which is rarely used in practice but is worth understanding for its conceptual value. The front-door criterion is useful when we cannot estimate the causal effect of *A* on *Y* and there is unmeasured confounding by *U*. Suppose further, that there is a mediator, *M*, that fully mediates the effect of *A* on *Y*. If *A* → *M* is unconfounded and *M* → *Y* is unconfounded, *A* → *Y* may be identified by estimating the separate identifiable paths through *M*. The front-door criterion is not widely used because requires measuring an appropriate mediator that fully captures the causal effect. However, understanding the front-door adjustment helps develop intuition for how estimating causal effects may be possible when there is unmeasured confounding (Pearl, 2009).

#### Pearl's structural causal models

In the potential outcomes framework, we represent interventions by setting variables to specific levels, e.g. setting the treatment to a specific value 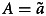. We have noted that counterfactual outcomes are conceived as the outcomes that would occur if, perhaps contrary to fact, an individual's treatment was set to a specific level. We use the convention *Y*_*i*_(*a*) or equivalently to denote the counterfactual or ‘potential’ outcome for individual *i* when that individual's treatment is set to *A*_*i*_ = *a*. Because we assume individual treatments to be independent and identically distributed (i.i.d.), we drop the subscripts when describing the potential outcomes for multiple individuals under specific levels of treatment. We denote the average of the potential outcomes as follows:
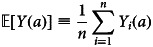


As noted above, we say that conditional exchangeability is satisfied if the potential outcomes are independent of the treatment assignment conditional on the measured covariates:
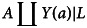


It is worth considering that causal directed acyclic graphs do not directly represent counterfactual outcomes. Instead, they evaluate whether causality can be identified from hypothetical interventions on the variables represented in a graph. Formally, causal directed acyclic graphs rely on Judea Pearl's do-calculus (Pearl, 2009), which relies on the concept of an ‘interventional distribution’. Under Pearl's do-calculus, any node in a graph can be intervened upon. Nodes and edges in a causal diagram correspond to non-parametric structural equations or what Pearl calls ‘structural causal models’ (Pearl, 2009). Note that non-parametric structural equations are causal-structural models. They are fundamentally different from statistical structural equation models that are employed in many human sciences. *Please do not confuse non-parametric structural equation models with statistical structural equation models* (VanderWeele, 2015). In a causal directed acyclic graph, non-parametric structural equations represent the underlying causal mechanisms without making specific parametric assumptions about the functional forms of relationships. It is important to note that non-parametric structural equation models, also known as structural causal models, are mathematical representations of the causal relationships between variables in a system. These equations describe the functional relationships between variables without specifying the particular functional form or the probability distributions of the variables. In contrast, statistical structural equation models, commonly used in the social sciences and psychology, make specific assumptions about the functional form of the relationships (e.g. linear, polynomial or exponential) and the probability distributions of the variables (e.g. normal, Poisson or binomial). Statistical structural equation models model observed data. Non-parametric structural equations state the assumed causal structure of the system – we do not use non-parametric structural equation models to do statistics. When we employ statistical structural equation models or any other statistical model, we must first state the assumed functional relationships that we maintain (under expert advice) hold for the data. We must do so without making assumptions about the functional form of the statistical model we will eventually employ – statistics come later, only after we have evaluated whether the causal effect we seek may be identified with data. Pearl's do-calculus and the rules of d-separation are based on non-parametric structural equations, which provide a flexible and generalisable framework for causal inference (Pearl, 2009).

Pearl's structural causal models work as follows.

Let *L* denote the common causes of treatment *A* and outcome *Y*:

- The node *L* in the corresponding DAG *G* corresponds to the non-parametric structural equation:*L* = *f*_*L*_(*U*_*L*_), where *f*_*L*_ is an unspecified function and *U*_*L*_ represents the exogenous error term or unmeasured factors affecting *L*.
- The treatment node *A* in *G* is associated with the non-parametric structural equation: *A* = *f*_*A*_(*L*, *U*_*A*_), where *f*_*A*_ is an unspecified function, *L* represents the common causes and *U*_*A*_ represents the exogenous error term or unmeasured factors affecting *A*.
- The outcome node *Y* in *G* is associated with the non-parametric structural equation: *Y* = *f*_*Y*_(*A*, *L*, *U*_*Y*_), where *f*_*Y*_ is an unspecified function, *A* represents the treatment, *L* represents the common causes and *U*_*Y*_ represents the exogenous error term or unmeasured factors affecting *Y*.

In Pearl's formalism, we assume that *U*_*L*_, *U*_*A*_ and *U*_*Y*_ are independent exogenous random variables. That is, we assume there are no direct arrows linking *A* to *Y* except through the common cause node *L*. Causal diagrams allow us to factorise the joint distribution of *L*, *A* and *Y* as a product of conditional probability distributions.

Define *O* as a distribution of independent and identically distributed observations such that *O* = (*L*, *A*, *Y*). The true distribution *P*_*O*_ is factorised as:
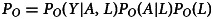
where *P*_*O*_(*L*) is the marginal distribution of the covariates *L*; *P*_*O*_(*A*|*L*) is the conditional distribution of the treatment given the covariates; and *P*_*O*_(*Y*|*A*, *L*) is the conditional distribution of the outcome given the treatment and covariates.

Pearl's do-calculus allows us to evaluate the consequences of intervening on variables represented in a causal DAG to interpret probabilistic dependencies and independencies in the conditional and marginal associations presented on a graph.

Here, we have developed counterfactual contrasts using the potential outcomes framework. The potential outcomes framework considers potential outcomes to be fixed and real (even if assigned non-deterministically). Pearl develops counterfactual contrasts using operations on structural functionals, referred to as ‘do-calculus’. In Pearl's framework, we obtain counterfactual inference by assuming that the nodes in a causal directed acyclic graph correspond to a system of structural equation models, such as those we just described.

Mathematically, potential outcomes and counterfactual interventions are equivalent, such that:
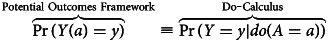
where the left-hand side of the equivalence is the potential outcomes framework formalisation of a potential outcome recovered by causal consistency, and the right-hand side is given by Pearl's do-calculus, which, as just mentioned, formalises interventional distributions on nodes of a graph that correspond to structural causal models.

In practice, whether one uses Pearl's do-calculus or the potential outcomes framework to interpret causal inferences is often irrelevant to identification results. However, there are theoretically interesting debates about edge cases. For example, Pearl's structural causal models permit the identification of contrasts that cannot be falsified under any experiment (Richardson & Robins, 2013a). Because advocates of non-parametric structural equation models treat causality as primitive, they are less concerned with the requirement for falsification (Díaz et al., 2021, 2023; Pearl, 2009; Rudolph et al., 2024). Additionally, the potential outcomes framework allows for identification in settings where the error terms in a structural causal model are not independent (Bulbulia, 2024b).

I have presented the potential outcomes framework because it is easier to interpret, more general, and – to my mind – clearer and more intellectually compelling (moreover, one does not need to be a verificationist to adopt it). However, for nearly every practical purpose, the do-calculus and ‘po-calculus’ (potential outcomes framework, refer to Shpitser and Tchetgen, 2016) are both mathematically and practically equivalent. And remember, the nodes and edges in a causal directed acyclic graph correspond to non-parametric structural equations: these equations represent causal mechanisms *without* making specific assumptions about the functional form of the assumed causal relationships encoded in the causal DAG. As currently employed, the statistical structural equation models used in human sciences often make implausible (or even incoherent) causal assumptions (Bulbulia, 2024b; VanderWeele, 2015). It is essential to draw a causal directed acyclic graph (causal DAG) before considering a statistical structural equation model.

### The five elementary structures of causality

Table 3 presents five elementary structures of causality from which all causal directed acyclic graphs are built. These elementary structures can be assembled in different combinations to clarify the causal relationships presented in a causal directed acyclic graph.

**Table 3.** The five elementary structures of causality from which all causal directed acyclic graphs can be built

Structure	Causal DAG	Explanation	Implication
*Two variables*			
1. Causality absent	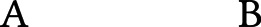	*A* and *B* have no causal effect on each other	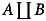
2. Causality present	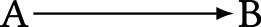	*A* causally affects *B*, and they are associated	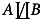
*Three variables*			
3. Fork	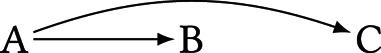	*A* causally affects both *B* and *C*; *B* and *C* are conditionally independent given *A*	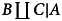
4. Chain	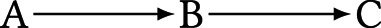	*C* is affected by *B* which is, in turn, affected by *A*; *A* and *C* are conditionally independent given *B*	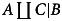
5. Collider	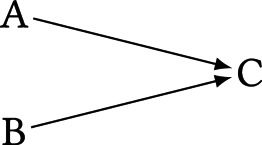	*C* is affected by both *A* and *B*, which are independent; conditioning on *C* induces association between *A* and *B*	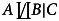

*Key*: A directed edge (arrow) denotes causal association. The absence of an arrow denotes no causal association. *Rules of d-separation:* In a causal diagram, a path is ‘blocked’ or ‘d-separated’ if a node along it interrupts causation. Two variables are d-separated if all paths connecting them are blocked or if there are no paths linking them, making them conditionally independent. Conversely, unblocked paths result in ‘d-connected’ variables, implying statistical association. Refer to Pearl (1995). Note that ‘d’ stands for ‘directional’.

*Implication*: 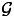 denotes a causal directed acyclic graph (causal DAG). *P* denotes a probability distribution function. Pearl proved that independence in a causal DAG 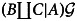 implies probabilistic independence 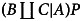; likewise if 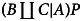 holds in all distributions compatible with 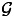, it follows that 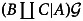 (refer to Pearl 2009: 61). We read causal graphs to understand the implications of causality for relationships in observable data. However, reading causal structures from data is more challenging because the relationships in observable data are typically compatible with more than one (and typically many) causal graphs.

### The five elementary rules for causal identification

Table 4 describes five elementary rules for identifying conditional independence using directed acyclic causal diagrams. There are no shortcuts to reasoning about causality. Each causal question must be asked in the context of a specific scientific question, and each causal graph must be built under the best lights of domain expertise. However, the following five elementary rules for confounding control are implied by the theorems that underpin causal directed acyclic graphs. They may be a useful start for evaluating the prospects for causal identification across a broad range of settings.

**Table 4.** Five elementary rules for causal identification

	Rule	Problem	Solution
1	**Ensure Causal Order**: Timing of *Aϕ*1 and *Yϕ*2 are incorrectly asserted; in truth, *Y*1 induces an association with *A*2	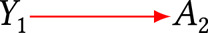	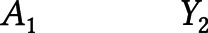
2	**Block back-door path by conditioning on a common cause or its proxy**: *A* and *Y* share both measured and unmeasured common causes; conditioning to block the open backdoor path	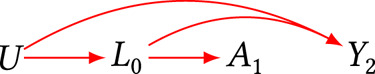	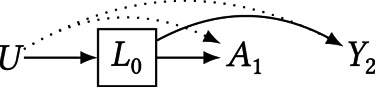
3	**Do not condition on a mediator**: 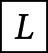 blocks the total causal effect of 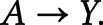 . If *L* may be affected by *A*, ensure *L* occurs before *A*	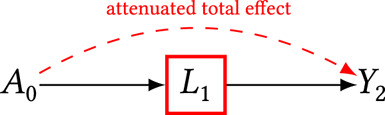	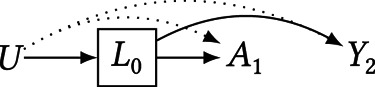
4	**Do not condition on a collider**: 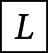 induces a non-causal association between *A* and *Y*. Ensure *L* occurs before *A* and that *A* occurs before *Y*	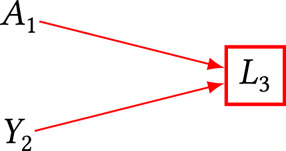	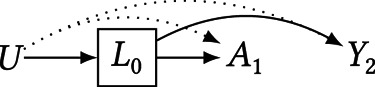
5	**Conditioning on a descendant is akin to conditioning on its parent**: 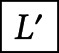 , a descendant of an unmeasured confounder *U*, may also be affected by *A*. Ensure *L*^′^ occurs before *A*. If *L*^′^ is not affected by *A* or *Y*, *L*^′^'s timing relative to *A* and *Y* is unimportant. We must only ensure that *U* occurs before *A* and that *A* occurs before *Y*	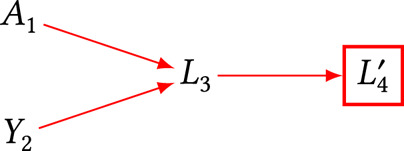	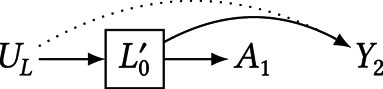

*Key*: *A* denotes the treatment; *Y* denotes the outcome; *U* denotes an unmeasured confounder; *L* denotes a confounder; 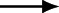 asserts causality; *t* subscript denotes the true relative timing of the variable; *ϕt* relative timing is asserted, here erroneously; 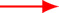 indicates a path for bias linking *A* to *Y* absent causation; 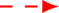 indicates a path for bias separating *A* and *Y*; 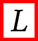 indicates that conditioning on *L* introduces bias (over-conditioning bias). We include 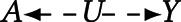 to clarify that we cannot typically be confident that all common causes of the treatment and outcome have been measured. Examples 1 and 3–5 illustrate how bias arises from erroneous variable timing: *ϕt* ≠ *t*.

1. *Ensure that treatments precede outcomes* – this rule is a logical consequence of our assumption that causality follows the arrow of time and that a causal directed acyclic graph is faithful to this ordering. However, the assumption that treatments precede outcomes may be easily violated where investigators cannot ensure the relative timing of events from their data.

Note that this assumption does not raise concerns in settings where past outcomes may affect future treatments. Indeed, an effective strategy for confounding control in such settings is to condition on past outcomes, and where relevant, on past treatments as well. For example, if we wish to identify the causal effect of *A*_1_ on *Y*_2_, and repeated-measures time series data are available, it may be useful to condition such that 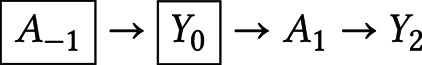. Critically, the relations of variables must be arranged sequentially without cycles.

Causal directed acyclic graphs must be acyclic. Yet most processes in nature include feedback loops. However, there is no contradiction as long as we represent these loops as sequential events. To estimate a causal effect of *Y* on *A*, we would focus on: 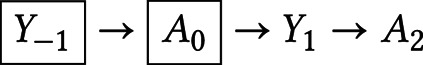. Departing from conventions we have previously used to label treatments and outcomes, here *Y* denotes the treatment and *A* denotes the outcome.

2. *Condition on common causes or their proxies* – this rule applies to settings in which the treatment *A* and the outcome *Y* share common causes. By conditioning on these common causes, we block the open backdoor paths that could introduce bias into our causal estimates. Controlling for these common causes (or their proxies) helps to isolate the specific effect of *A* on *Y*. Note that we do not draw a path from *A* → *Y* in this context because it represents an interventional distribution. In a causal directed acyclic graph, conditioning does not occur on interventional distributions. We do not box *A* and *Y*.

3. *Do not condition on a mediator when estimating total effects* – this rule applies to settings in which the variable *L* is a mediator of *A* → *Y*. Recall that Pearl's backdoor path criterion requires that we do not condition on a descendant of the treatment. Here, conditioning on *L* violates the backdoor path criterion, risking bias for a total causal effect estimate. We must not condition on a mediator if we are interested in total effect estimates. Note we draw the path from *A* → *Y* to underscore that this specific overconditioning threat occurs in the presence of a true treatment effect. Over-conditioning bias can operate in the absence of a true treatment effect. This is important because conditioning on a mediator might create associations without causation. In many settings, ensuring accuracy in the relative timing of events in our data will prevent the self-inflicted injury of conditioning on a common effect of the treatment.

4. *Do not condition on a collider* – this rule applies to settings in which *L* is a common effect of *A* and *Y*. Conditioning on a collider may invoke a spurious association. Again, the backdoor path criterion requires that we do not condition on a descendant of the treatment. We would not be tempted to condition on *L* if we knew that it was an effect of *A*. In many settings, ensuring accuracy in the relative timing of events in our data will prevent the self-inflicted injury of conditioning on a common effect of the treatment and outcome.

5. *Proxy rule: conditioning on a descendant is akin to conditioning on its parent* – this rule applies to settings where *L*^′^ is an effect from another variable *L*. The graph considers when *L*^′^ is downstream of a collider. Here again, in many settings, ensuring accuracy in the relative timing of events in our data will prevent the self-inflicted injury of conditioning on a common effect of the treatment and outcome.

### Summary Part 2

We use causal directed acyclic graphs to represent and evaluate structural sources of bias. We do not use these causal graphs to represent the entirety of the causal system in which we are interested, but rather *only those features necessary to evaluate conditional exchangeability*, or equivalently to evaluate d-separation. Moreover, causal directed acyclic graphs should not be confused with the structural equation models employed in the statistical structural equation modelling traditions (refer also to Rohrer et al., 2022). To repeat, although Pearl's formalism is built upon ‘Non-Parametric Structural Equation Models’, the term ‘Structural Equation Model’ can be misleading. Causal directed acyclic graphs are structural models that represent assumptions about reality, they are not statistical models. We use structural causal models to evaluate identifiability. We create causal graphs before we embark on statistical modelling. They aim to clarify how to write statistical models by elucidating which variables we must include in our statistical models and, equally important, and which variables we must exclude to avoid invalidating our causal inferences. All causal graphs are grounded in our assumptions about the structures of causation. Although it is sometimes possible – under assumptions – to automate causal discovery (Peters et al., 2016) we cannot fully dispense with assumption because the causal structures of the world are underdetermined by the data (Quine, 1981; J. M. Robins, 1999).

The distinction between structural and statistical models is fundamental because in the absence of clearly defined causal contrasts on well-defined treatments, well-defined outcomes and well-defined populations, and absent carefully evaluated assumptions about structural sources of bias in the relationship between treatments and outcomes, the statistical structural equation modelling tradition offers no guarantees that the coefficients investigators recover are interpretable. Misunderstanding this difference between structural and statistical models has led to considerable confusion across the human sciences (Bulbulia, 2022, 2024b; VanderWeele, 2015, 2022; VanderWeele & Vansteelandt, 2022).

## Part 3: how causal directed acyclic graphs clarify the importance of timing of events recorded in data

As noted in the previous section, the five elementary rules of confounding control reveal the importance of ensuring accurate timing in the occurrence of the variables whose structural features a causal directed acyclic graph encodes. We begin by considering seven examples of confounding problems resolved when accuracy in the timing of the occurrence of variables is ensured. These examples refer to causal graphs in Table 5. We use the symbol *G* to denote a graph. We use the convention: 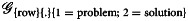 to indicate a causal directed acyclic graph in the table.

**Table 5.** Causal DAGs illustrate how ensuring the relative timing of the occurrence of variables of interest addresses common forms of bias when estimating causal effects

Examples of confounding bias avoided with accurate temporal order			
	Bias	Problem	Accuracy in timing
1	*Reverse causation* – incorrectly asserted *Aϕ*1 and *Yϕ*2; in truth *Y*1 causes association with *A*2	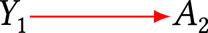	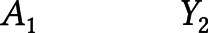
2	*Confounding by common cause* – an unmeasured variable is assumed to cause both *A* and *Y*	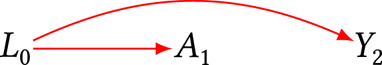	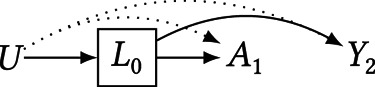
3	*Mediator bias* – incorrect timing asserted by *Lϕ*, leading to controlling for a mediator, which distorts the true causal effect	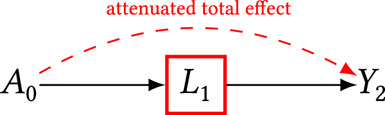	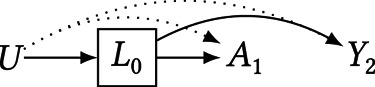
4	*Collider bias* – incorrect timing asserted by *Lϕ*1, leading to controlling for a collider, creating a non-causal association between *A* and *Y*	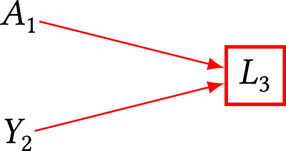	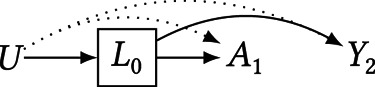
5	*Collider proxy bias* – conditioning on a descendant of a collider introduces bias	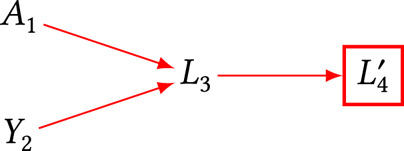	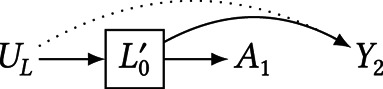
6	*Post-treatment collider stratification bias* – conditioning on a variable affected by treatment, even if this is not a mediator, may induce bias	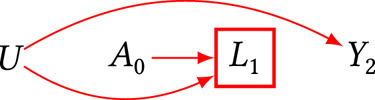	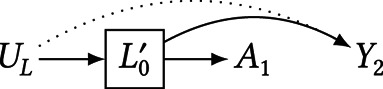
7	*Unmeasured common cause* – conditioning on baseline exposure and outcome provides powerful confounding control and recovers incident exposure effect	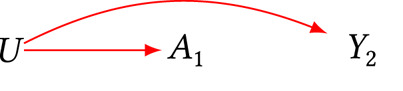	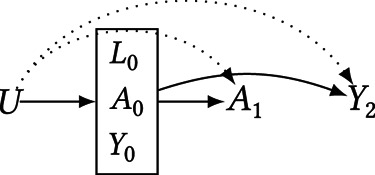

*Key*: *A* denotes the treatment; *Y* denotes the outcome; *U* denotes an unmeasured confounder; *L* denotes a confounder; 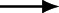 denotes causal edge; *k* subscript denotes the true relative timing of the variable; *ϕk* relative timing is asserted, here erroneously; 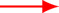 indicates a path for bias linking *A* to *Y* absent causation; 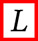 indicates a path for bias separating *A* and *Y* from conditioning on a mediator; 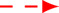 indicates that conditioning on *L* that introduces bias (over-conditioning bias). We include 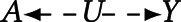 to clarify that we cannot typically be confident that all common causes of the treatment and outcome have been measured.

*Example 1, reverse-causation* – *ϕ* timing in the exposure and outcome is incorrect.

*Examples 3–6*, asserted timing *ϕ* of confounder is incorrect: *L*_*ϕ*0_ ≠ *L*_0_.

*Example 7* shows how we can reduce unmeasured confounding by conditioning on baseline values of the exposure and outcome.

### Example 1: reverse causation

Table 5 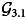 illustrates bias from reverse causation. Suppose we are interested in the causal effect of marriage on well-being. If we observe that married people are happier than unmarried people, we might erroneously infer that marriage causes happiness, or happiness causes marriage (refer to McElreath, 2020).

Table 5 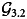 clarifies a response. Ensure that the treatment is observed before the outcome is observed. Note further that the treatment, in this case, is not clearly specified because ‘marriage’ is unclear. There are at least four causal contrasts we might consider when thinking of ‘marriage’, namely:

*Y*(0,0) – the potential outcome when there is no marriage.

*Y*(0,1) – the potential outcome when there is a shift to marriage from no marriage.

*Y*(1,0) – the potential outcome under divorce.

*Y*(1,1) – the potential outcome from marriage prevalence.

Each of these four outcomes may be contrasted with the others, yielding six unique contrasts. Which do we wish to consider? ‘What is the causal effect of marriage on happiness?’ is ill-defined. This question does not uniquely state which of the six causal contrasts to consider. The first step in causal inference is to state a well-defined causal question in terms of interventions and outcomes to be compared. For a worked example refer to Bulbulia (2024b).

### Example 2: confounding by common cause

Table 5 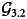 illustrates confounding by common cause. Suppose there is a common cause, *L*, of the treatment, *A*, and outcome,*Y*. In this setting, *L* may create a statistical association between *A* and *Y*, implying causation in its absence. Most human scientists will be familiar with the threat to inference in this setting: a ‘third variable’ leads to a statistical association between treatment and outcome absent causation.

Suppose that smoking, *L*, is a common cause of both yellow fingers, *A*, and cancer,*Y*. Here, *A* and *Y* may show an association without causation. If investigators were to scrub the hands of smokers, this would not affect cancer rates.

Table 5 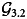 clarifies a response. Condition on the common cause, smoking. Within strata of smokers and non-smokers, there will be no association between yellow fingers and cancer.

### Example 3: mediator bias

Table 5 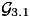 illustrates mediator bias. Conditioning on the effect of treatment blocks the flow of information from treatment to outcome, biasing the total effect estimate.

Suppose investigators are interested in whether cultural ‘beliefs in big Gods’ *A* affect social complexity *Y*. Suppose that ‘economic trade’, *L*, is both a common cause of the treatment and outcome. To address confounding by a common cause, we must condition on economic trade. However, timing matters. If we condition on measurements that reflect economic trade after the emergence of beliefs in big Gods, we may bias our total effect estimate.

Table 5 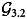 clarifies a response. Ensure that measurements of economic trade are obtained for cultural histories before big Gods arise. Do not condition on post-treatment instances of economic trade.

### Example 4: collider bias

Table 5 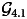 illustrates collider bias. Imagine a randomised experiment investigating the effects of different settings on individuals’ self-rated health. In this study, participants are assigned to either civic settings (e.g. community centres) or religious settings (e.g. places of worship). The treatment of interest, *A*, is the type of setting, and the outcome, *Y*, is self-rated health. Suppose there is no effect of setting on self-rated health. However, suppose both setting and rated health independently influence a third variable: cooperativeness. Specifically, imagine religious settings encourage cooperative behaviour, and at the same time, individuals with better self-rated health are more likely to engage cooperatively. Now suppose the investigators decide to condition on cooperativeness, which in reality is the common effect of *A* and the outcome *Y*. Their rationale might be to study the effects of setting on health among those who are more cooperative or perhaps to ‘control for’ cooperation in the health effects of religious settings. By introducing such ‘control’, the investigators would inadvertently introduce collider bias, because the control variable is a common effect of the treatment and the outcome. If both *A* and *Y* are positively associated with *L*, *A* and *Y* will be negatively associated with each other. However, such an association is a statistical artefact. Were we to intervene on *A*, *Y* would not change.

Table 5 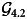 clarifies a response. If the worry is that cooperativeness is a confounder, ensure that cooperativeness is measured before the initiation of exposure to religious settings.

### Example 5: collider proxy bias

Table 5 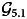 illustrates bias from conditioning on the proxy of a collider. Consider again the scenario described in Example 4: collider bias, but instead of controlling for cooperativeness, investigators control for charitable donations, a proxy for cooperativeness. Here, because the control variable is a descendant of a collider, conditioning on the proxy of the collider is akin to conditioning on the collider itself.

Table 5 *G*_5.2_ clarifies a response. Do not condition on charitable donations, an effect of treatment.

### Example 6: post-treatment collider stratification bias

Table 5 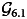 illustrates post-treatment collider stratification bias. Consider again an experiment investigating the effect of religious service on self-rated health. Suppose we measure ‘religiosity’ after the experiment, along with other demographic data. Suppose further that religious setting affects religiosity, as does an unmeasured confounder, such as childhood deprivation. Suppose that childhood deprivation affects self-reported health. Although our experiment ensured randomisation of the treatment and thus ensured no unmeasured common causes of the treatment and outcome, conditioning on the post-treatment variable ‘religiosity’ opens a back-door path from the treatment to the outcome. This path is *A*_0_ 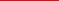 *L*_1_ 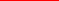 *U* 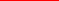 *Y*_2_. We introduced confounding into our randomised experiment.

Table 5 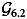 clarifies a response. Do not condition on a variable that the treatment may affect (refer to Cole et al. (2010) for a discussion of theoretical examples; refer to Montgomery et al. (2018) for evidence of the widespread prevalence of post-treatment adjustment in published political science experiments; refer also to Bulbulia (2024d)).

### Example 7: conditioning on past treatments and past outcomes to control for unmeasured confounders

Table 5 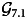 illustrates the threat of unmeasured confounding. In ‘real world’ studies, this threat is ubiquitous. Table 5 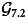 clarifies a response. With at least three repeated measurements, investigators may greatly reduce unmeasured confounding by controlling for past measurements of the treatment as well as past measurements of the outcome. With such control, any unmeasured confounder must be orthogonal to its effects at baseline (refer to VanderWeele et al., 2020). Moreover, controlling for past treatments allows investigators to estimate an incident exposure effect over a prevalence exposure effect. The prevalence exposure effect describes the effect of current or ongoing exposures (treatments) on outcomes. This effect risks leading to erroneous conclusions. The incident exposure effect targets initiation into treatment, which is typically the effect we obtain from experiments. To obtain the incident exposure effect, we generally require that events in the data can be accurately classified into at least three relative time intervals (refer to Hernán et al., 2016a; Danaei et al., 2012; VanderWeele et al., 2020; Bulbulia, 2022).

### Summary Part 3

The examples in Part 3 reveal that the ability to order treatments, outcomes, and their common causes on a timeline is necessary for obtaining valid inferences. When timing is ensured, we can use Pearl's backdoor path adjustment algorithm to evaluate identification, subject to the assumptions encoded in a causal directed acyclic graph.

## Part 4: how causal directed acyclic graphs clarify the insufficiency of ensuring the timing of events recorded in data for causal identification

We next present a series of illustrations that clarify ordering variables in time is insufficient insurance against confounding biases. All graphs in Part 4 refer to Table 6.

**Table 6.** Common confounding scenarios in which ordering of variable timing is insufficient for causal identification

Examples of bias not resolved by sequential data collection			
	Bias	Problem	Response
1	*M-bias*: over-conditioning bias. Response: do not condition on *L*.	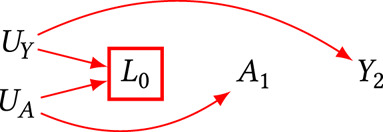	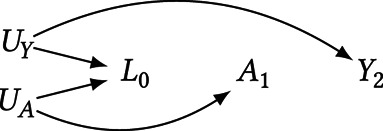
2	*M-bias, L is a confounder*: pre-treatment collider *L* is also a confounder. *Response*: adjust for proxies of unmeasured confounders that do not induce M-bias.	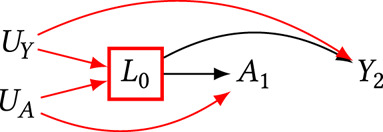	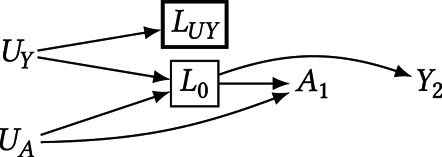
3	*Unmeasured confounding Response*: unless a proxy of an unmeasured confounder is a mediator or collider, conditioning on this proxy will reduce bias even if the proxy occurs after the outcome.	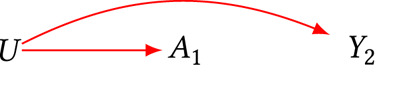	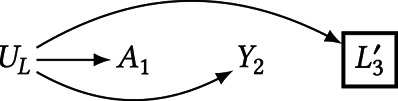
4	*Residual confounding.* *Response*: sensitivity analysis.	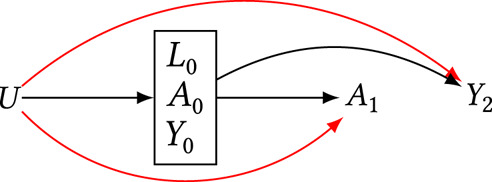	Sensitivity analysis
5	*Treatment affects confounder of mediator/outcome path.* *Response*: estimate controlled direct effect.	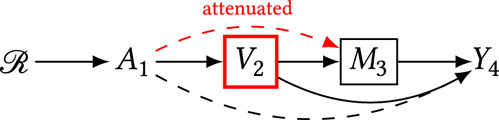	Natural direct/indirect effects are not identified; consider estimating the mediated effects of *V* and *M* combined, or an analogue of the desired estimand using random draws from distributions that are not observed (‘recanting twins’)
6	*Collider stratification bias without confounder feedback*: no effect of treatment on future confounder yet time-varying confounding from collider stratification bias. *Response*: special estimators; requires strong sequential ignorability	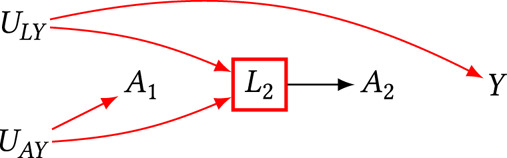	Special estimators; strong sequential ignorability
*Key*: 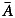 denotes the treatment, or sequential treatments: for *k* ∈ *K* measurement intervals {*As* < *p*,*Ap*;*s*, *p* ∈ *K*}; *Y*, the outcome;*U*, unmeasured confounder;*L*, measured confounder;*L*^′^, proxy for an unmeasured confounder;*M*^′^, mediator of 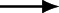 ; *V*, intermediary mediator; , denotes randomised to treatment assignment; 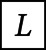 indicates conditioning on variable *X* eliminates or reduces; 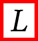 indicates that conditioning on *L* introduces bias. 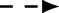 , mediated direct effect; 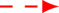 , bias for total effect of *A* on *Y* from conditioning on a mediator; 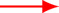 indicates a path for bias linking *A* to *Y* absent causation.			

### Example 1: M-bias

Table 6 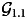 illustrates the threat of over-conditioning on pre-treatment variables – ‘M-bias’. Suppose we want to estimate the effect of religious service attendance on charitable donations. We obtain time-series data and include a rich set of covariates, including baseline measures of religious service and charity. Suppose there is no treatment effect. Suppose further that we condition on loyalty measures, yet loyalty affects neither religious service attendance nor charitable giving. However, imagine that loyalty is affected by two unmeasured confounders. Furthermore, imagine that one's childhood upbringing (an unmeasured variable) affects both loyalty and inclinations to religious service but not charitable giving. *U*_*A*_ denotes this unmeasured confounder. Furthermore, suppose wealth affects loyalty and charitable giving but not religious service. *U*_*Y*_ denotes this unmeasured confounder. In this setting, because loyalty is a collider of the unmeasured confounders, conditioning on loyalty opens a path between treatment and outcome. This path is 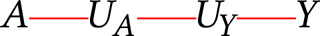.

Table 6 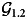 clarifies a response. If we are confident that 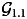 describes the structural features of confounding, we should not condition on loyalty.

### Example 2: M-bias where the pre-treatment collider is a confounder

Table 6 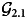 illustrates the threat of incorrigible confounding. Imagine the scenario in 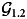 and 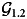 but with one change. Loyalty is indeed a common cause of religious service attendance (the treatment) and charitable giving (the outcome). If we do not condition on loyalty, we have unmeasured confounding. This is bad. If we condition on loyalty, as we have just considered, we also have unmeasured confounding. This is also bad.

Table 6 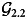 clarifies a response. Suppose that although we have not measured wealth, we have measured a surrogate of wealth, say neighbourhood deprivation. Conditioning on this surrogate is akin to conditioning on the unmeasured confounder; we should adjust for neighbourhood deprivation.

### Example 3: opportunities for post-treatment conditioning for confounder control

Table 6 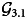 illustrates the threat of unmeasured confounding. Suppose we are interested in whether curiosity affects educational attainment. The effect might be unclear. Curiosity might increase attention but it might also increase distraction. Consider an unmeasured genetic factor *U* that influences both curiosity and educational attainment, say anxiety. Suppose we do not have early childhood measures of anxiety in our dataset. We have unmeasured confounding. This is bad.

Table 6 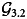 clarifies a response. Suppose *U* also affects melanin production in hair follicles. If grey hair is an effect of a cause of curiosity, and if grey hair cannot be an effect of educational attainment, we could diminish unmeasured confounding by adjusting for grey hair in adulthood. This example illustrates how conditioning on a variable that occurs after the treatment has occurred, or even after the outcome has been observed, may prove useful for confounding control. When considering adjustment strategies, it is sometimes useful to consider adjustment on post-treatment confounders; however, it must be clear that the confounder is not affected by the treatment.

### Example 4: residual confounding after conditioning on past treatments and past outcomes

Table 6 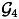 illustrates the threat of confounding even after adjusting for baseline measures of the treatment and the outcome. Imagine that childhood deprivation, an unmeasured variable, affects both religious service attendance and charitable giving. Despite adjusting for religious status and charitable giving at baseline, childhood deprivation might influence changes in one or both variables over time. This can create a longitudinal association between religious service attendance and charitable giving without a causal relationship. Strictly speaking, the causal effect cannot be identified. We may estimate an effect and perform sensitivity analyses to check how much unmeasured confounding would be required to explain way an effect (refer to Linden et al., 2020); we may also seek negative controls (refer to Hernán & Robins, 2024).

### Example 5: intermediary confounding in causal mediation

Table 6 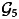 illustrates the threat of treatment confounding in causal mediation. Imagine that the treatment is randomised; there is no treatment-outcome confounding. Nor is there treatment-mediator confounding. 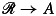 ensures that backdoor paths from the treatment to the outcome are closed. We may obtain biased results despite randomisation because the mediator is not randomised. Suppose we are interested in whether the effects of COVID-19 lockdowns on psychological distress were mediated by levels of satisfaction with the government. Suppose that assignment to COVID-19 lockdowns was random, and that time series data taken before COVID-19 provides comparable population-level contrasts. Despite random assignment to treatment, assume that there are variables that may affect both satisfaction with the government and psychological distress. For example, job security or relationship satisfaction might plausibly function as common causes of the mediator (government satisfaction) and the outcome (psychological distress). To obtain valid inference for the mediator-outcome path, we must control for these common causes.

Table 6 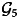 reveals the difficulty in decomposing the total effect of COVID-19 on psychological distress into the direct effect of COVID-19 that is not mediated by satisfaction with the government and the indirect effect that is mediated. Let us assume that confounders of the mediator–outcome path are themselves potentially affected by the treatment. In this example, imagine that COVID-19 lockdowns affect relationship satisfaction because couples are trapped in ‘captivity’. Imagine further that COVID-19 lockdowns affect job security, which is reasonable if one owns a street-facing business. If we adjust for these intermediary variables along the path between the treatment and outcome, we will partially block the treatment–mediator path. This means that we will not be able to obtain a natural indirect effect estimate that decomposes the effect of the treatment into that part that goes through the intermediary path *A* 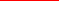 *V* 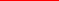 *M* 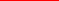 *Y* and that part that goes through the mediated path independently of *V*, namely *A* 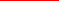 *V* 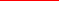 *M* 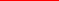 *Y*. However, it may be possible to estimate controlled direct effects – that is, direct effects when the mediator is fixed to different levels (Greenland et al., 1999; Shpitser et al., 2022; VanderWeele, 2015), or to obtain approximations of the natural direct effect (Bulbulia, 2024b; refer to Díaz et al., 2023; Stensrud et al., 2023).

### Example 6: treatment confounder feedback in sequential treatments

Table 6 illustrates the threat of treatment confounder feedback in sequential treatment regimes. Suppose we are interested in whether beliefs in big Gods affect social complexity. Suppose that beliefs in big Gods affect economic trade and that economic trade may affect beliefs in big Gods and social complexity. Suppose the historical record is fragmented such that there are unmeasured variables that affect both trade and social complexity. Even if these unmeasured variables do not affect the treatment, conditioning on the *L* (a confounder) and sequential treatment opens a backdoor path *A* 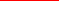 *L* 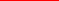 *U* 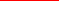 *Y*. We have confounding.

Table 6 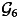 reveals the difficulty of sequentially estimating causal effects. To estimate an effect requires special estimators under the assumption of sequential randomisation for fixed treatments and the assumption of strong sequential randomisation for time-varying treatments – that is, for treatments whose present levels depend on the levels of past treatments and and measured confounders affected by those treatments (Díaz et al., 2021; Haneuse & Rotnitzky, 2013; Hernán et al., 2004; Hoffman et al., 2023; Richardson & Robins, 2013a; J. Robins, 1986; Rotnitzky et al., 2017; Van Der Laan & Rose, 2011, 2018; Williams & Díaz, 2021; Young et al., 2014).

Importantly, we have six potential contrasts for the two sequential treatments: beliefs in big Gods at both time points vs. beliefs in big Gods at neither time point; beliefs in big Gods first, then lost vs. never believing in big Gods at both. We can compute six causal contrasts for these four fixed regimens, as shown in Table 7.

**Table 7.** Table outlines four fixed treatment regimens and six causal contrasts in time-series data where treatments vary over time

Type	Description	Counterfactual outcome
Regime	Always believe in big Gods	*Y*(1, 1)
Regime	Never believe in big Gods	*Y*(0, 0)
Regime	Believe once first, then scepticism	*Y*(1, 0)
Regime	Start with scepticism, then believe	*Y*(0, 1)
Contrast	Always believe vs. Never believe	*E*[*Y*(1, 1) − *Y*(0, 0)]
Contrast	Always believe vs. Treat once first	*E*[*Y*(1, 1) − *Y*(1, 0)]
Contrast	Always believe vs. Treat once second	*E*[*Y*(1, 1) − *Y*(0, 1)]
Contrast	Never believe vs. Treat once first	*E*[*Y*(0, 0) − *Y*(1, 0)]
Contrast	Never believe vs. Treat once second	*E*[*Y*(0, 0) − *Y*(0, 1)]
Contrast	Believe once first vs. Believe once second	*E*[*Y*(1, 0) − *Y*(0, 1)]

A limitation of directed acyclic causal diagrams is that we do not project factorisations of the counterfactual contrasts onto the graphs themselves. Evaluating counterfactual identification, using Single World Intervention Graphs can be helpful (Richardson & Robins, 2013b, 2023; J. M. Robins & Richardson, 2010). I consider intermediate confounding in more detail in Bulbulia (2024b).

### Example 7: collider stratification bias in sequential treatments

Table 6 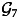 illustrates the threat of confounding bias in sequential treatments even without treatment–confounder feedback. Assume the setting is 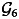 with two differences. First, assume that the treatment, beliefs in big Gods, does not affect trade networks. However, assume that an unmeasured confounder affects both the beliefs in big Gods and the confounder, trade networks. Such a confounder might be openness to outsiders, a feature of ancient cultures for which no clear measures are available. We need not imagine that treatment affects future states of confounders for time-varying confounding. It would be sufficient to induce bias for an unmeasured confounder to affect the treatment and the confounder, in the presence of another confounder that affects both the confounder and the outcome.

Table 6 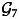 reveals the challenges of sequentially estimating causal effects. Yet again, to estimate causal effects here requires special estimators, under the assumption of sequential randomisation for fixed treatments, and the assumption of strong sequential randomisation for time-varying treatments (Díaz et al., 2021; Haneuse & Rotnitzky, 2013; Hernán et al., 2004; Hoffman et al., 2023; Richardson & Robins, 2013a; J. Robins, 1986; Rotnitzky et al., 2017; Van Der Laan & Rose, 2011, 2018; Williams & Díaz, 2021; Young et al., 2014). We note again that a specific causal contrast must be stated, and we must ask which cultures our causal effect estimates generalise to.

Readers should be aware that merely applying currently popular tools of time-series data analysis – multi-level models and structural equation models – will not overcome the threats of confounding in sequential treatments. Applying models to data will not recover consistent causal effect estimates. Again, space constraints prevent us from discussing statistical estimands and estimation here (refer to Bulbulia, 2024a).

### Summary Part 4

Directed acyclic graphs reveal that ensuring the timing of events in one's data does not ensure identification. In some cases, certain mediated effects cannot be identified by any data, as we discussed in the context of mediation analysis with intermediate confounding. However, across the human sciences, we often apply statistical models to data and interpret the outputs as meaningful. **Causal diagrams show that standard statistical modelling practices, including those in structural equation modelling, readily invite misleading causal conclusions.**

## Part 5: creating causal diagrams: pitfalls and tips

The primary interest of causal diagrams is to address *identification problems*. Pearl's backdoor adjustment theorem proves that if we adopt an adjustment set such that *A* and *Y* are d-separated, and furthermore do not condition on a variable along the path from *A* to *Y*, then association is causation.

Here is how investigators may construct safe and effective directed acyclic graphs.

### 1. Clarify the causal question and target population

An identification strategy is relative to the question at hand. The adjustment criteria for estimating an effect of *A* on *Y* will generally differ from those for estimating an effect of *Y* on *A*. Before attempting to draw any causal diagram, state the problem your diagram addresses and the population to whom it applies. Additionally, when adopting a specific identification strategy for a treatment or set of treatments, the coefficients we obtain for the other variables in the model will often be biased causal effect estimates for those variables.

Moreover, the *coefficients obtained from statistical models developed to estimate causal effects will typically not have a causal interpretation* (Chatton et al., 2020; Cole & Hernán, 2008; VanderWeele, 2009). This implication has wide-ranging consequences for scientific reporting. For example, if regression coefficients are reported at all, they should come with clear warnings against interpreting them as having any causal meaning or interpretation (McElreath, 2020; Westreich & Greenland, 2013). Powerful machine learning algorithms treat these parameters as a nuisance, and in many cases, coefficients cannot be obtained. Referees of human science journals need to be alerted to this fact and retrained accordingly.

### 2. Consider whether the three fundamental assumptions for causal inference may be satisfied

Merely possessing data, even if the data are richly detailed time-series data, does not mean our causal questions will find answers. Along with identification, we must also consider the causal consistency and positivity assumptions, refer to Part 1.

### 3. Clarify the meanings of symbols and conventions

It is fair to say that the state of terminology in causal inference is a dog's breakfast (for a glossary, refer to supplement S1). Meanings and conventions vary not only for terminology but also for causal graphical conventions. For example, whereas we have denoted unmeasured confounders using the variable *U*, those who follow Pearl will often draw a bi-directional arrow. Although investigators will have their preferences, there is generally little substantive interest in one's conventions, only that they are made clear, frequently repeated (as I have done repeatedly in the key for each graph table) and applied correctly.

### 4. Include all common causes of the treatment and outcome

Once we have stated our causal question, we are ready to create a draft of our causal graph. This graph should incorporate the most recent common causes (parents) of both the treatment and the outcome, or, where measures are not available, measures for available proxies.

Where possible, aggregate functionally similar common causes and label them with a single node. For example, all baseline confounders that are a common cause of the treatment and outcome might be labelled *L*_0_. Time-varying confounders might be labelled *L*_1_, *L*_2_, …*L*_*τ*−1_, where 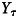 is the outcome at the end of study.

How do we determine whether a variable is a common cause of the treatment and the outcome? We might not always be in a position to know. Remember that a causal DAG asserts structural assumptions. Expertise in crafting causal diagrams does not guarantee expertise in encoding plausible structural assumptions! Therefore, creating and revising causal DAGs should involve topic specialists. Additionally, the decision-making processes should be thoroughly documented in published research, even if this documentation is placed in supplementary materials.

### 5. Consider potential unmeasured confounders

We leverage domain expertise not only to identify measured sources of confounding but also – and perhaps most importantly – to identify potential unmeasured confounders. These should be included in our causal diagrams. Because we cannot guard against all unmeasured confounding, it is essential to perform sensitivity analyses and to consider developing multiple analytic strategies to provide multiple channels of evidence for the question at hand, such as instrumental variables, negative control treatments, negative control outcomes and mendelian randomisation (Angrist & Pischke, 2009; Smith et al., 2022).

### 6. Ensure that the causal directed acyclic graph is acyclic and practice good chronological hygiene

Although not strictly necessary, it may be useful to annotate the temporal sequence of events using subscripts (e.g. *L*_0_, *A*_1_,*Y*_2_), as we have done here. Moreover, it is a great help to your audience (and to yourself) to spatially order your directed acyclic graph to reflect the progression of causality in time – either left-to-right or top-to-bottom. What might be called ‘chronological hygiene’ will considerably enhance comprehensibility, and allow you to spot errors you might otherwise miss – such as worrying about whether a post-treatment variable is a confounder (it is not) – and we should not condition on an effect of the treatment if our interest is in a total treatment effect. Note there are other post-treatment biases to worry about, such as directed measurement error bias (Bulbulia, 2024c); however, it is perilous to fix such biases through adjustment.

### 7. Represent paths structurally, not parametrically

Whether a path is linear is unimportant for causal identification – and remember causal diagrams are tools for causal identification. Focus on whether paths exist, not their functional form (linear, non-linear, etc.).

Consider a subway map of Paris. We do not include all the streets on this map, all noteworthy sites or a detailed overview of the holdings by room in the Louvre. We use other maps for these purposes. Remember, the primary function of a causal diagram is to ensure d-separation. If a causal diagram is to be useful, it must remove almost every detail about the reality it assumes.

### 8. Minimise paths to those necessary for addressing an identification problem

Reduce clutter; only include paths critical for a specific question (e.g. backdoor paths, mediators). For example, in Table 6 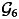 and Table 6 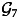, I did not draw arrows from the first treatment to the second treatment. Although I assume that such arrows exist, drawing them was not, in these examples, relevant to evaluating the identification problem at hand.

### 9. When temporal order is unknown, explicitly represent this uncertainty on your causal diagram

In many settings, the relevant timing of events cannot be ascertained with confidence. To address this, we adopt the convention of indexing nodes with uncertain timing using *X*_*ϕt*_ notation. Although there is no widely adopted convention for representing uncertainty in timing, our primary obligation is to be clear.

### 10. Create, report and deploy multiple graphs

Causal inference hinges on assumptions, and experts might disagree. When the structure of reality encoded in a causal graph is uncertain or debated, investigators should produce multiple causal diagrams that reflect these uncertainties and debates.

By stating different assumptions and adopting multiple modelling strategies that align with these assumptions, we might find that our causal conclusions are robust despite differences in structural assumptions. Even when different structural assumptions lead to opposing causal inferences, this knowledge can guide future data collection to resolve these differences. The primary goal of causal inference, as with all science, is to truthfully advance empirical understanding. Assertions are poor substitutes for honesty. Rather than asserting a single causal directed graph, investigators should follow the implications of several.

### 11. Use automated identification algorithms such as daggity with care

Automated software can assist with identification tasks, such as factorising complex conditional independencies. However, automated software may not converge on identifying the optimal set of confounders in the presence of intractable confounding.

Consider Tyler VanderWeele's *modified disjunctive cause criterion*. VanderWeele (2019) recommends obtaining a maximally efficient adjustment, termed a ‘confounder set’. A member of this set is any variable that can reduce or remove structural sources of bias. The strategy is as follows:

1. Control for any variable that causes the treatment, the outcome, or both.
2. Control for any proxy of an unmeasured variable that is a shared cause of both the treatment and outcome.
3. Define an instrumental variable as a variable associated with the treatment but not influencing the outcome independently, except through the treatment. Exclude any instrumental variable that is not a proxy for an unmeasured confounder from the confounder set (VanderWeele, 2019).

VanderWeele's modified disjunctive cause criterion is an excellent strategy for selecting an optimal confounder set. However, this set might not remove all structural sources of confounding bias in most observational settings. As such, an automated algorithm might reject it. This rejection could be unwise because, in non-randomised treatment assignments, we should nearly always include relations of unmeasured confounding in our causal graphs, as I have done throughout this article. Rejecting causal inferences in observational settings entirely because one suspects unmeasured confounders would be imprudent. We should nearly always suspect unmeasured confounders. Nevertheless, there are many instances where observational causal inferences have been found to closely approximate randomised controlled trails (Hernán et al., 2008b, 2016b; Hernán & Robins, 2006b).

For example, consider Table 6 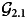, where we encountered intractable confounding. What if there were no proxy for an unmeasured confounder? Should we condition on the measured confounder and induce M-bias, leave the backdoor path from the measured confounder open or not attempt causal inferences at all? The answer depends on assumptions about the relative strength of confounding in the causal diagram. Rather than relying on a generic strategy, robust causal inference requires subject-specialist expertise (Smith et al., 2022).

### 12. Clarify assumptions about structural bias from measurement error and target population restriction (also known as ‘selection bias’)

Space constraints prevented us from examining how causal directed acyclic graphs can clarify structural biases from measurement error and restrictions of the target population in the sample population at the start and end of the study. We can (and should) examine structural features of bias in these settings. For an overview, refer to Bulbulia (2024c).

## Conclusions

### Limitations

*First, I have focused on the application of causal diagrams to confounding bias; however, there are other biases that threaten causal inference besides confounding biases.* Causal directed acyclic graphs can also be extended to evaluate measurement-error biases and some features of target population restriction bias (also called ‘selection restriction bias’). Valid causal inferences require addressing all structural sources of bias. This work does not aim for complete coverage of how causal diagrams may be useful for off-label applications other than assessing d-separation, but it hopes to stimulate curiosity (Bulbulia, 2024c; Hernán & Robins, 2024; Hernán, 2017; Hernán & Cole, 2009; Liu et al., 2023; VanderWeele & Hernán, 2012).

*Second, I have not reviewed other graphical tools for identification, such as Single World Intervention Graphs*. Although causal directed acyclic graphs are powerful tools for addressing identification problems, they are not the only graphical tools researchers use to investigate causality. For example, J. Robins (1986) developed the ‘finest fully randomised causally interpreted structured tree graph (FFRCISTG)’, which has been more recently revived and simplified in Single World Intervention Graphs (refer to Richardson & Robins, 2013b). These graphs explicitly factorise counterfactual states, which can be helpful for identification in complex longitudinal settings. For some, representing counterfactual states on a graph is more satisfying, as it allows inspection of the conditional independence of expectations over *Y*(a*) and *Y*(*a*) separately. Refer to Bulbulia (2024b) for use cases.

*Third, I have not reviewed workflows downstream of causal identification*. This article does not cover statistical estimands, statistical estimation, and the interpretation and reporting of causal inferences, which come downstream of causal graphs in causal inference workflows. Rapid developments in machine learning offer applied researchers new tools for handling model misspecification (Díaz et al., 2021; Hoffman et al., 2023; Laan & Gruber, 2012; Van Der Laan & Rose, 2018; Williams & Díaz, 2021) and assessing treatment effect heterogeneity (Athey et al., 2019; Athey & Wager, 2021; Vansteelandt & Dukes, 2022; Wager & Athey, 2018). Those interested in workflows for causal inference in panel studies might consider VanderWeele et al. (2020). The workflows in my research group can be found here: Bulbulia (2024a). For general approaches, I recommend: https://tlverse.org/tmle3/ (accessed 10 June 2024). However, readers should be aware that workflows for statistical designs and estimation are evolving quickly.

Nevertheless, after precisely stating our causal question, the most difficult and important challenge is considering whether and how it might be identified in the data. The ‘statistical models first’ approach routinely applied in most human sciences is soon ending. This approach has been attractive because it is relatively easy to implement – the methods do not require extensive training – and because the application of statistical models to data appears rigorous. However, if the coefficients we recover from these methods have meaning, this is typically accidental. Without a causal framework, these coefficients are not just uninformative about what works and why (Ogburn & Shpitser, 2021).

There are many good resources available for learning causal directed acyclic graphs (Barrett, 2021; Cinelli et al., 2022; Greenland et al., 1999, 1999; Hernán & Robins, 2024; Major-Smith, 2023; McElreath, 2020; Morgan & Winship, 2014; Pearl, 2009; Rohrer, 2018; Suzuki et al., 2020). This work aims to add to these resources, first by providing additional conceptual orientation to the frameworks and workflows of causal data science, highlighting the risks of applying causal graphs without this understanding; second, by using causal diagrams to emphasise the importance of ensuring relative timing for the variables whose causal relationships are represented on the graph; and third, by employing causal diagrams to clarify the limitations of longitudinal data for certain questions in causal mediation and time-varying confounding under time-varying treatments, which remain topics of confusion in many human sciences (see Bulbulia, 2024b for a detailed explanation).

For those just getting started on causal diagrams, I recommend Miguel Hernán's free course at https://www.edx.org/learn/data-analysis/harvard-university-causal-diagrams-draw-your-assumptions-before-your-conclusions (accessed 10 June 2024).

For those seeking a slightly more technical but still accessible introduction to causal inference and causal DAGs, I recommend Brady Neal's introduction to causal inference course and textbook, both freely available at https://www.bradyneal.com/causal-inference-course (accessed 10 June 2024).

### Neurath's boat: on the priority of assumptions in science

We might wonder, if not from the data, where do our assumptions about causality come from? We have said that our assumptions must come from expert knowledge. Our reliance on expert knowledge might seem counterintuitive for building scientific knowledge. Should we not use data to build scientific knowledge, not the other way around? Is scientific history not a record of expert opinions being undone?

The Austrian philosopher Otto Neurath famously described scientific progress using the metaphor of a ship that must be rebuilt at sea:

every statement about any happening is saturated with hypotheses of all sorts and these in the end are derived from our whole world-view. We are like sailors who on the open sea must reconstruct their ship but are never able to start afresh from the bottom. Where a beam is taken away a new one must at once be put there, and for this the rest of the ship is used as support. In this way, by using the old beams and driftwood, the ship can be shaped entirely anew, but only by gradual reconstruction. (Neurath, 1973, p. 199)

Neurath emphasises the iterative process of accumulating scientific knowledge; new insights are formed from the foundation of existing knowledge (Godfrey-Smith, 2006, 2009; Quine, 1981).

Causal diagrams are at home in Neurath's boat. We should resist the tradition of science that believes that knowledge develops solely from the results of statistical tests applied to data. The data have never fully contained the answers we seek. When reconstructing knowledge, we have always relied on assumptions. Causal graphs enable us to make these assumptions explicit and to understand what we obtain based on them.

## Supporting information

Bulbulia supplementary material Bulbulia supplementary material

## Research transparency and reproducibility

No data were used in this manuscript

## References

[ref1] Angrist, J. D., & Pischke, J.-S. (2009). Mostly harmless econometrics: An empiricist's companion. Princeton University Press.

[ref2] Athey, S., & Wager, S. (2021). Policy learning with observational data. Econometrica, 89(1), 133–161. 10.3982/ECTA15732

[ref3] Athey, S., Tibshirani, J., & Wager, S. (2019). Generalized random forests. The Annals of Statistics, 47(2), 1148–1178. 10.1214/18-AOS1709

[ref4] Bareinboim, E., & Pearl, J. (2013). A general algorithm for deciding transportability of experimental results. Journal of Causal Inference, 1(1), 107–134.

[ref5] Barrett, M. (2021). Ggdag: Analyze and create elegant directed acyclic graphs. https://CRAN.R-project.org/package=ggdag

[ref6] Bulbulia, J. A. (2022). A workflow for causal inference in cross-cultural psychology. Religion, Brain & Behavior, 13(3), 291–306. 10.1080/2153599X.2022.2070245

[ref7] Bulbulia, J. A. (2024a). A practical guide to causal inference in three-wave panel studies. *PsyArXiv Preprints*. 10.31234/osf.io/uyg3d

[ref8] Bulbulia, J. A. (2024b). Methods in causal inference. Part 2: Interaction, mediation, and time-varying treatments. Evolutionary Human Sciences, 6. 10.1017/ehs.2024.32 PMC11588565 39600621

[ref10] Bulbulia, J. A. (2024c). Methods in causal inference. Part 3: Measurement error and external validity threats. Evolutionary Human Sciences, 6. 10.1017/ehs.2024.33 PMC11588564 39600618

[ref9] Bulbulia, J. A. (2024d). Methods in causal inference. Part 4: Confounding in experiments. Evolutionary Human Sciences, 6. 10.1017/ehs.2024.34 PMC11658928 39703944

[ref11] Bulbulia, J. A., Afzali, M. U., Yogeeswaran, K., & Sibley, C. G. (2023). Long-term causal effects of far-right terrorism in New Zealand. PNAS Nexus, 2(8), pgad242. 10.1093/pnasnexus/pgad242 PMC10443658 37614668

[ref12] Chatton, A., Le Borgne, F., Leyrat, C., Gillaizeau, F., Rousseau, C., Barbin, L., …, Foucher, Y. (2020). G-computation, propensity score-based methods, and targeted maximum likelihood estimator for causal inference with different covariates sets: A comparative simulation study. Scientific Reports, 10(1), 9219. 10.1038/s41598-020-65917-x 32514028 PMC7280276

[ref13] Cinelli, C., Forney, A., & Pearl, J. (2022). A crash course in good and bad controls. Sociological Methods &Research, 00491241221099552. 10.1177/00491241221099552

[ref14] Cole, S. R., & Hernán, M. A. (2008). Constructing inverse probability weights for marginal structural models. American Journal of Epidemiology, 168(6), 656–664. 18682488 10.1093/aje/kwn164 PMC2732954

[ref15] Cole, S. R., Platt, R. W., Schisterman, E. F., Chu, H., Westreich, D., Richardson, D., & Poole, C. (2010). Illustrating bias due to conditioning on a collider. International Journal of Epidemiology, 39(2), 417–420. 10.1093/ije/dyp334 19926667 PMC2846442

[ref16] Dahabreh, I. J., & Hernán, M. A. (2019). Extending inferences from a randomized trial to a target population. European Journal of Epidemiology, 34(8), 719–722. 10.1007/s10654-019-00533-2 31218483

[ref17] Dahabreh, I. J., Robins, J. M., Haneuse, S. J., & Hernán, M. A. (2019). Generalizing causal inferences from randomized trials: Counterfactual and graphical identification. *arXiv Preprint arXiv:*1906.10792.

[ref18] Danaei, G., Tavakkoli, M., & Hernán, M. A. (2012). Bias in observational studies of prevalent users: lessons for comparative effectiveness research from a meta-analysis of statins. American Journal of Epidemiology, 175(4), 250–262. 10.1093/aje/kwr301 22223710 PMC3271813

[ref19] Díaz, I., Hejazi, N. S., Rudolph, K. E., & Der Laan, M. J. van. (2021). Nonparametric efficient causal mediation with intermediate confounders. Biometrika, 108(3), 627–641.

[ref20] Díaz, I., Williams, N., & Rudolph, K. E. (2023). Journal of Causal Inference, 11(1), 20220077. 10.1515/jci-2022-0077

[ref21] Godfrey-Smith, P. (2006). The strategy of model-based science. Biology and Philosophy, 21, 725–740.

[ref22] Godfrey-Smith, P. (2009). Theory and reality: An introduction to the philosophy of science. University of Chicago Press.

[ref23] Greenland, S. (2003). Quantifying biases in causal models: Classical confounding vs collider-stratification bias. Epidemiology, 300–306. 12859030

[ref24] Greenland, S., Pearl, J., & Robins, J. M. (1999). Causal diagrams for epidemiologic research. Epidemiology *(*Cambridge, Mass*.)*, 10(1), 37–48. 9888278

[ref25] Greifer, N. (2023). WeightIt: Weighting for covariate balance in observational studies.

[ref26] Greifer, N., Worthington, S., Iacus, S., & King, G. (2023). Clarify: Simulation-based inference for regression models. https://iqss.github.io/clarify/

[ref27] Haneuse, S., & Rotnitzky, A. (2013). Estimation of the effect of interventions that modify the received treatment. Statistics in Medicine, 32(30), 5260–5277. 23913589 10.1002/sim.5907

[ref28] Hernán, M. A. (2017). Invited commentary: Selection bias without colliders. American Journal of Epidemiology, 185(11), 1048–1050. 10.1093/aje/kwx077 28535177 PMC6664806

[ref29] Hernán, M. A., & Cole, S. R. (2009). Invited commentary: Causal diagrams and measurement bias. American Journal of Epidemiology, 170(8), 959–962. 10.1093/aje/kwp293 19755635 PMC2765368

[ref30] Hernán, M. A., & Robins, J. M. (2006a). Estimating causal effects from epidemiological data. Journal of Epidemiology & Community Health, 60(7), 578–586. 10.1136/jech.2004.029496 16790829 PMC2652882

[ref31] Hernán, M. A., & Robins, J. M. (2006b). Estimating causal effects from epidemiological data. Journal of Epidemiology & Community Health, 60(7), 578–586. 10.1136/jech.2004.029496 16790829 PMC2652882

[ref32] Hernán, M. A., & Robins, J. M. (2024). Causal inference: What if? Taylor & Francis. https://www.hsph.harvard.edu/miguel-hernan/causal-inference-book/

[ref33] Hernán, M. A., Hernández-Díaz, S., & Robins, J. M. (2004). A structural approach to selection bias. Epidemiology, 15(5), 615–625. https://www.jstor.org/stable/20485961 15308962 10.1097/01.ede.0000135174.63482.43

[ref34] Hernán, M. A., Alonso, A., Logan, R., Grodstein, F., Michels, K. B., Willett, W. C., …, Robins, J. M. (2008a). Observational studies analyzed like randomized experiments: An application to postmenopausal hormone therapy and coronary heart disease. Epidemiology, 19(6), 766. 10.1097/EDE.0b013e3181875e61 18854702 PMC3731075

[ref35] Hernán, M. A., Alonso, A., Logan, R., Grodstein, F., Michels, K. B., Willett, W. C., …, Robins, J. M. (2008b). Observational studies analyzed like randomized experiments: An application to postmenopausal hormone therapy and coronary heart disease. Epidemiology, 19(6), 766. 10.1097/EDE.0b013e3181875e61 18854702 PMC3731075

[ref36] Hernán, M. A., Sauer, B. C., Hernández-Díaz, S., Platt, R., & Shrier, I. (2016a). Specifying a target trial prevents immortal time bias and other self-inflicted injuries in observational analyses. Journal of Clinical Epidemiology, 79, 70–75. 27237061 10.1016/j.jclinepi.2016.04.014 PMC5124536

[ref37] Hernán, M. A., Sauer, B. C., Hernández-Díaz, S., Platt, R., & Shrier, I. (2016b). Specifying a target trial prevents immortal time bias and other self-inflicted injuries in observational analyses. Journal of Clinical Epidemiology, 79, 70–75. 27237061 10.1016/j.jclinepi.2016.04.014 PMC5124536

[ref38] Hernán, M. A., Wang, W., & Leaf, D. E. (2022). Target trial emulation: A framework for causal inference from observational data. JAMA, 328(24), 2446–2447. 10.1001/jama.2022.21383 36508210

[ref39] Hernán, M. A., Robins, J. M., et al. (2017). Per-protocol analyses of pragmatic trials. N Engl J Med, 377(14), 1391–1398. 28976864 10.1056/NEJMsm1605385

[ref40] Hoffman, K. L., Salazar-Barreto, D., Rudolph, K. E., & Díaz, I. (2023). Introducing longitudinal modified treatment policies: A unified framework for studying complex exposures. 10.48550/arXiv.2304.09460 39109818

[ref41] Holland, P. W. (1986). Statistics and causal inference. Journal of the American Statistical Association, 81(396), 945–960.

[ref42] Hume, D. (1902). Enquiries concerning the human understanding: And concerning the principles of morals. Clarendon Press.

[ref43] Imai, K., King, G., & Stuart, E. A. (2008). Misunderstandings between experimentalists and observationalists about causal inference. Journal of the Royal Statistical Society Series A: Statistics in Society, 171(2), 481–502.

[ref44] Laan, M. J. van der, & Gruber, S. (2012). Targeted minimum loss based estimation of causal effects of multiple time point interventions. The International Journal of Biostatistics, 8(1). 10.1515/1557-4679.1370 22611591

[ref45] Lash, T. L., Rothman, K. J., VanderWeele, T. J., & Haneuse, S. (2020). Modern epidemiology. Wolters Kluwer. https://books.google.co.nz/books?id=SiTSnQEACAAJ 10.1007/s10654-021-00778-w PMC8416883 34216355

[ref46] Lauritzen, S. L., Dawid, A. P., Larsen, B. N., & Leimer, H.-G. (1990). Independence properties of directed Markov fields. Networks, 20(5), 491–505.

[ref47] Lewis, D. (1973). Causation. The Journal of Philosophy, 70(17), 556–567. 10.2307/2025310

[ref48] Lin, W. (2013). Agnostic notes on regression adjustments to experimental data: Reexamining Freedman's critique. The Annals of Applied Statistics, 7(1), 295–318. 10.1214/12-AOAS583

[ref49] Linden, A., Mathur, M. B., & VanderWeele, T. J. (2020). Conducting sensitivity analysis for unmeasured confounding in observational studies using e-values: The evalue package. The Stata Journal, 20(1), 162–175.

[ref50] Liu, Y., Schnitzer, M. E., Herrera, R., Díaz, I., O'Loughlin, J., & Sylvestre, M.-P. (2023). The application of target trials with longitudinal targeted maximum likelihood estimation to assess the effect of alcohol consumption in adolescence on depressive symptoms in adulthood. American Journal of Epidemiology, kwad241. 10.1093/aje/kwad241 38061692

[ref51] Major-Smith, D. (2023). Exploring causality from observational data: An example assessing whether religiosity promotes cooperation. Evolutionary Human Sciences, 5, e22. 37587927 10.1017/ehs.2023.17 PMC10426067

[ref52] McElreath, R. (2020). Statistical rethinking: A Bayesian course with examples in R and Stan. CRC press.

[ref53] Montgomery, J. M., Nyhan, B., & Torres, M. (2018). How conditioning on posttreatment variables can ruin your experiment and what to do about it. American Journal of Political Science, 62(3), 760–775. 10.1111/ajps.12357

[ref54] Morgan, S. L., & Winship, C. (2014). Counterfactuals and causal inference: Methods and principles for social research (2nd ed.). Cambridge University Press. 10.1017/CBO9781107587991

[ref55] Neal, B. (2020). Introduction to causal inference from a machine learning perspective. Course lecture notes (draft). https://www.bradyneal.com/Introduction_to_Causal_Inference-Dec17_2020-Neal.pdf

[ref56] Neurath, O. (1973). Anti-spengler. In M. Neurath & R. S. Cohen (Eds.), Empiricism and sociology (pp. 158–213). Springer Netherlands. 10.1007/978-94-010-2525-6_6

[ref57] Ogburn, E. L., & Shpitser, I. (2021). Causal modelling: The two cultures. Observational Studies, 7(1), 179–183. 10.1353/obs.2021.0006

[ref58] Pearl, J. (1988). Probabilistic reasoning in intelligent systems: Networks of plausible inference. Morgan Kaufmann.

[ref59] Pearl, J. (1995). Causal diagrams for empirical research. Biometrika, 82(4), 669–688.

[ref60] Pearl, J. (2009). Causality. Cambridge University Press.

[ref61] Pearl, J., & Bareinboim, E. (2022). External validity: From do-calculus to transportability across populations (1st ed., Vol. 36, pp. 451–482). Association for Computing Machinery. 10.1145/3501714.3501741

[ref62] Peters, J., Bühlmann, P., & Meinshausen, N. (2016). Causal inference by using invariant prediction: Identification and confidence intervals. Journal of the Royal Statistical Society Series B: Statistical Methodology, 78(5), 947–1012.

[ref63] Quine, W. V. O. (1981). Theories and things. Harvard University Press.

[ref64] Richardson, T. S., & Robins, J. M. (2013a). Single world intervention graphs: A primer. https://core.ac.uk/display/102673558

[ref65] Richardson, T. S., & Robins, J. M. (2013b). Single world intervention graphs: A primer. Second UAI Workshop on Causal Structure Learning, Bellevue, WA. https://citeseerx.ist.psu.edu/document?repid=rep1&type=pdf&doi=07bbcb458109d2663acc0d098e8913892389a2a7

[ref66] Richardson, T. S., & Robins, J. M. (2023). Potential outcome and decision theoretic foundations for statistical causality. Journal of Causal Inference, 11(1), 20220012.

[ref67] Robins, J. (1986). A new approach to causal inference in mortality studies with a sustained exposure period – application to control of the healthy worker survivor effect. Mathematical Modelling, 7(9–12), 1393–1512.

[ref68] Robins, J. M. (1999). Association, causation, and marginal structural models. Synthese, 121(1/2), 151–179.

[ref69] Robins, J. M., & Richardson, T. S. (2010). Alternative graphical causal models and the identification of direct effects. Causality and Psychopathology: Finding the Determinants of Disorders and Their Cures, 84, 103–158.

[ref70] Rohrer, J. M. (2018). Thinking clearly about correlations and causation: Graphical causal models for observational data. Advances in Methods and Practices in Psychological Science, 1(1), 27–42.

[ref71] Rohrer, J. M., Hünermund, P., Arslan, R. C., & Elson, M. (2022). That's a lot to process! Pitfalls of popular path models. Advances in Methods and Practices in Psychological Science, 5(2). 10.1177/25152459221095827

[ref72] Rotnitzky, A., Robins, J., & Babino, L. (2017). On the multiply robust estimation of the mean of the g-functional. https://arxiv.org/abs/1705.08582

[ref73] Rubin, D. B. (1976). Inference and missing data. Biometrika, 63(3), 581–592. 10.1093/biomet/63.3.581

[ref74] Rudolph, K. E., Williams, N. T., & Diaz, I. (2024). Practical causal mediation analysis: Extending nonparametric estimators to accommodate multiple mediators and multiple intermediate confounders. Biostatistics, kxae012. 10.1093/biostatistics/kxae012 PMC11471960 38576206

[ref75] Shpitser, I., & Tchetgen, E. T. (2016). Causal inference with a graphical hierarchy of interventions. Annals of Statistics, 44(6), 2433. 28919652 10.1214/15-AOS1411 PMC5597261

[ref76] Shpitser, I., Richardson, T. S., & Robins, J. M. (2022). Multivariate counterfactual systems and causal graphical models. In Probabilistic and causal inference: The works of Judea Pearl (pp. 813–852).

[ref77] Smith, G. D., Richmond, R. C., & Pingault, J.-B. (2022). Combining human genetics and causal inference to understand human disease and development. Cold Spring Harbor Laboratory Press.

[ref78] Stensrud, M. J., Robins, J. M., Sarvet, A., Tchetgen Tchetgen, E. J., & Young, J. G. (2023). Conditional separable effects. Journal of the American Statistical Association, 118(544), 2671–2683.

[ref79] Stuart, E. A., Bradshaw, C. P., & Leaf, P. J. (2015). Assessing the generalizability of randomized trial results to target populations. Prevention Science, 16(3), 475–485. 10.1007/s11121-014-0513-z 25307417 PMC4359056

[ref80] Stuart, E. A., Ackerman, B., & Westreich, D. (2018). Generalizability of randomized trial results to target populations: Design and analysis possibilities. Research on Social Work Practice, 28(5), 532–537. 30034203 10.1177/1049731517720730 PMC6049838

[ref81] Suzuki, E., Shinozaki, T., & Yamamoto, E. (2020). Causal diagrams: Pitfalls and tips. Journal of Epidemiology, 30(4), 153–162. 10.2188/jea.JE20190192 32009103 PMC7064555

[ref82] Van Der Laan, M. J., & Rose, S. (2011). Targeted learning: Causal inference for observational and experimental data. Springer. https://link.springer.com/10.1007/978-1-4419-9782-1

[ref83] Van Der Laan, M. J., & Rose, S. (2018). Targeted learning in data science: Causal inference for complex longitudinal studies. Springer International. http://link.springer.com/10.1007/978-3-319-65304-4

[ref84] VanderWeele, T. J. (2009). Marginal structural models for the estimation of direct and indirect effects. Epidemiology, 18–26. 19234398 10.1097/EDE.0b013e31818f69ce

[ref85] VanderWeele, T. J. (2012). Confounding and effect modification: Distribution and measure. Epidemiologic Methods, 1(1), 55–82. 10.1515/2161-962X.1004 25473593 PMC4249691

[ref86] VanderWeele, T. J. (2015). Explanation in causal inference: Methods for mediation and interaction. Oxford University Press.

[ref87] VanderWeele, T. J. (2019). Principles of confounder selection. European Journal of Epidemiology, 34(3), 211–219. 30840181 10.1007/s10654-019-00494-6 PMC6447501

[ref88] VanderWeele, T. J. (2022). Constructed measures and causal inference: Towards a new model of measurement for psychosocial constructs. Epidemiology, 33(1), 141. 10.1097/EDE.0000000000001434 34669630 PMC8614532

[ref89] VanderWeele, T. J., & Ding, P. (2017). Sensitivity analysis in observational research: Introducing the E-value. Annals of Internal Medicine, 167(4), 268–274. 10.7326/M16-2607 28693043

[ref90] VanderWeele, T. J., & Hernán, M. A. (2012). Results on differential and dependent measurement error of the exposure and the outcome using signed directed acyclic graphs. American Journal of Epidemiology, 175(12), 1303–1310. 10.1093/aje/kwr458 22569106 PMC3491975

[ref91] VanderWeele, T. J., & Vansteelandt, S. (2022). A statistical test to reject the structural interpretation of a latent factor model. Journal of the Royal Statistical Society Series B: Statistical Methodology, 84(5), 2032–2054. 36818188 10.1111/rssb.12555 PMC9937555

[ref92] VanderWeele, T. J., Mathur, M. B., & Chen, Y. (2020). Outcome-wide longitudinal designs for causal inference: A new template for empirical studies. Statistical Science, 35(3), 437–466.

[ref93] Vansteelandt, S., & Dukes, O. (2022). Assumption-lean inference for generalised linear model parameters. Journal of the Royal Statistical Society Series B: Statistical Methodology, 84(3), 657–685.

[ref94] Wager, S., & Athey, S. (2018). Estimation and inference of heterogeneous treatment effects using random forests. Journal of the American Statistical Association, 113(523), 1228–1242. 10.1080/01621459.2017.1319839

[ref95] Webster-Clark, M., & Breskin, A. (2021). Directed acyclic graphs, effect measure modification, and generalizability. American Journal of Epidemiology, 190(2), 322–327. 32840557 10.1093/aje/kwaa185

[ref96] Westreich, D., & Cole, S. R. (2010). Invited commentary: Positivity in practice. American Journal of Epidemiology, 171(6). 10.1093/aje/kwp436 PMC2877454 20139125

[ref97] Westreich, D., & Greenland, S. (2013). The table 2 fallacy: Presenting and interpreting confounder and modifier coefficients. American Journal of Epidemiology, 177(4), 292–298. 23371353 10.1093/aje/kws412 PMC3626058

[ref98] Westreich, D., Edwards, J. K., Lesko, C. R., Stuart, E., & Cole, S. R. (2017). Transportability of trial results using inverse odds of sampling weights. American Journal of Epidemiology, 186(8), 1010–1014. 10.1093/aje/kwx164 28535275 PMC5860052

[ref99] Westreich, D., Edwards, J. K., Lesko, C. R., Cole, S. R., & Stuart, E. A. (2019). Target validity and the hierarchy of study designs. American Journal of Epidemiology, 188(2), 438–443. 30299451 10.1093/aje/kwy228 PMC6357801

[ref100] Williams, N. T., & Díaz, I. (2021). lmtp: Non-parametric causal effects of feasible interventions based on modified treatment policies. 10.5281/zenodo.3874931

[ref101] Young, J. G., Hernán, M. A., & Robins, J. M. (2014). Identification, estimation and approximation of risk under interventions that depend on the natural value of treatment using observational data. Epidemiologic Methods, 3(1), 1–19. 25866704 10.1515/em-2012-0001 PMC4387917

