# Supplementary material for: Methods in causal inference. Part 1: causal diagrams and confounding
Source: Evol Hum Sci. 2024 Sep 27;6:e40. doi: 10.1017/ehs.2024.35 (PMC11588567; doi:10.1017/ehs.2024.35)
Supplement: Bulbulia supplementary material [file S2513843X24000355sup001.pdf]

Supplementary files for “Methods in Causal Inference Part 1: Causal Diagrams and Confounding”

Joseph A. Bulbulia[1]

[1] Victoria University of Wellington, New Zealand ORCID 0000-0002-5861-2056

2024-06-20

Table of contents

S1. Glossary 2
S2. Causal Inference in History: The Difficulty in Satisfying the Three Fundamental Assumptions for Causal Inference 4
S3. Causal Consistency Under Multiple Versions of Treatment 6
References 8

List of Tables

1 Glossary 3

S1. Glossary

Table 1: Glossary

Term	Definition
**Acyclic**	No variable can be an ancestor or descendant of itself on a causal graph.
**Adjacent Nodes**	Two nodes connected by an arrow are adjacent.
**Adjustment Set**	Variables conditioned to block all backdoor paths between treatment (A) and outcome (Y).
**Ancestor/Descendants**	Nodes connected by directed edges. All descendants of an ancestor can be reached by directed paths.
**Arrow**	Represents direct causation in a causal diagram, pointing from cause to effect.
**Average Treatment Effect (ATE)**	The difference in expected outcomes between treated and untreated units across a specified population. Synonym for Marginal Effect.
**Backdoor Path**	Path that, if not blocked, may associate the treatment and outcome without causality.
**Causal Contrast**	The difference in expected outcomes under different treatment levels.
**Causal Contrast Scale**	The metric for quantifying causal contrasts, chosen based on outcome type and research question.
**Causal Diagram (Causal DAG)**	A graph representing causal relationships to evaluate an identification problem; must be acyclic and describe all confounding, measured and unmeasured for the target population.
**Causal Estimand**	The causal contrast of interest in a study; specifies the intervention, outcome, contrast scale, and target population; stated before analysis.
**Causal Path**	Asserts a change in the parent node will induce a change in its child.
**Censoring**	the sample population is not representative of the target population at baseline (left censoring) or is no longer representative at the end of study (right censoring).
**Collider/"Immorality"**	A variable where two causal paths meet head-to-head, may induce non-causal associations between its parents.
**Conditional Average Treatment Effect (CATE)**	The treatment effect for specific subgroups, defined by measured characteristics.
**Conditioning**	Adjustment for variables in analysis to distinguish causal effects from associations.
**Confounding**	Treatment and outcome are associated independently of causality or are disassociated despite causality, relative to the causal question.
**Confounder**	A variable or set of variables form part of an ideal identification strategy to reduce or eliminate confounding.
**Counterfactual or Potential outcomes**	Hypothetical outcomes under different treatment conditions to be contrasted, only one may be realised for each observed unit.
**Direct Effect (Natural Direct Effect)**	The difference between potential outcomes when the treatment is applied and the mediator is set to no-treatment versus when neither the treatment nor the mediator is applied.
***d*-separation**	Backdoor paths are blocked, satisfying the assumption of 'no unmeasured confounding'.
**Descendant (Child)**	A node causally influenced by a prior node (Parent). A child is a parent's direct descendant.
**Effect-Measure Modifier/Effect-Modifier**	A variable that affects the magnitude or direction of a causal effect.
**Estimator**	Algorithm to compute a statistical estimand from data.
**External Validity/Target Validity**	The generalisability of study findings to the prespecified target population; assumes internal validity.
**Factorisation**	Decomposing the joint probability distribution of variables into a product of conditional probabilities of each variable given its parents.
**Heterogeneous Treatment Effects**	Variation in treatment effects across subgroups or contexts.
**Identification Problem**	Ensure no unmeasured confounding.
**Incident Exposure Effect**	Causal effect of initiating a new treatment.
**Indirect Effect (Natural Indirect Effect)**	The average difference in potential outcomes when the mediator is at its natural value under treatment versus no treatment.
**Instrumental Variable**	Associated with treatment but affecting the outcome only through the treatment, used for estimating causal effects amidst confounding.
**Intention-to-Treat Effect**	The effect of treatment assignment, what random assignment obtains.
**Internal Validity**	The extent to which causal associations in the study population are accurately identified.
**Inverse Probability of Censoring Weights**	Weights used to adjust for bias due to attrition in longitudinal studies.
**Inverse Probability of Treatment Weights**	Weights that create a pseudo-population to achieve treatment balance across conditions.
**Local Markov Assumption**	assumption that a variable is independent of its non-descendants given its immediate parents in a causal graph.
**Longitudinal Study/Panel Study**	A research design that repeatedly tracks and measures the same units over time.
**Loss-to-follow-up**	Participant attrition.
**Markov Assumption**	assumption that a variable is independent of its non-descendants given its parents in a causal graph
**Marginal Effect**	Synonym for Average Treatment Effect.
**Measurement Error Bias**	Bias introduced when measurements of variables are inaccurately recorded, either through correlated or direct measurement errors, or when uncorrelated errors mask the true effects.
**Mediator**	A variable through which a treatment affects an outcome.
**Modularity Assumption**	Interventions on one set of variables do not directly alter the conditional distribution of other variables, given their direct causes.
**Node**	Represents a variable in a causal diagram, also called "Vertex"
**Observational Study**	Treatment assignment is not controlled by the investigator.
**Parent/Child**	Adjacent nodes connected by a directed path.
**Path**	Nodes are connected by a sequence of edges. Directed paths follow directed edges.
**Per-Protocol Effect**	The causal effect under full-treatment adherence.
**Prevalent Exposure Effect**	Effect of current or ongoing treatments.
**Propensity Score**	The probability of receiving a treatment based on observed characteristics used for confounding adjustment in observational studies.
**Randomised Treatment Assignment**	Chance treatment assignment.
**Randomised Controlled Trial (RCT)**	Uses random treatment assignment to balance confounders across the treatments to be compared.
**Reverse Causation**	Mistaking the effect for the cause in an analysis.
**Sample Weights**	Adjusts sample data to represent the target population in analysis better.
**Selection Bias**	Systematic errors from non-representative study participation or attrition affecting generalisability.
**Sequentially Treatment**	multiple treatments may be fixed our time-varying
**Single World Intervention Graph (SWIG)**	A graph to obtain causal identification under a single counterfactual treatment regime by splitting nodes into random and fixed components, where the fixed inherits edges directed into the node (parents) and the random inherits edges out (children).
**Single World Intervention Template (SWIT)**	A graph-valued function or template generates SWIGs (is not itself a graph).
**Statistical Estimand**	The parameter of interest in a statistical model, not necessarily causal.
**Statistical Estimate**	The value obtained for a statistical estimand from data analysis.
**Statistical Model**	Describes covariance between variables; without structural assumptions, statistical models do not identify causal effects.
**Structural Model**	Assumptions about causal relationships encoded in diagrams, essential for identifying causality from statistical associations.
**Study Population**	The population from which data are collected, also called the "sample population."
**Target Population**	The broader population to which study results are intended to apply.
**Target Trial**	An observational study emulating an ideal experiment by pre-specifying a causal estimand, eligibility criteria, and data ordering for an incident exposure effect.
**Time-Varying Confounding**	Confounding that changes over time, complicating causal effect estimation using standard methods.
**Total Effect**	The difference in mean potential outcomes under contrasted treatments in a study.

S2. Causal Inference in History: The Difficulty in Satisfying the Three Fundamental Assumptions for Causal Inference

Consider the Protestant Reformation of the 16th century, which initiated religious change throughout much of Europe. Historians have argued that Protestantism caused social, cultural, and economic changes in those societies where it took hold (see: Weber (1905); Weber (1993); Swanson (1967); Swanson (1971); Basten & Betz (2013), and for an overview, see: Becker et al. (2016)).

Suppose we want to estimate the Protestant Reformation's 'Average Treatment Effect'. Let $A = a^*$ denote the adoption of Protestantism. We compare this effect with that of remaining Catholic, represented as $A = a$. We assume that both the concepts of 'adopting Protestantism' and 'economic development' are well-defined (e.g., GDP +1 century after a country has a Protestant majority contrasted with remaining Catholic). The causal effect for any individual country is $Y_i(a^*) - Y_i(a)$. Although we cannot identify this effect, if the basic assumptions of causal inference are met, we can estimate the average or marginal effect by conditioning on the confounding effects of L:

$$ATE_{\text{economic development}} = \mathbb{E}[Y(\text{Became Protestant}|L) - Y(\text{Remained Catholic}|L)]$$

When asking causal questions about the economic effect of adopting Protestantism versus remaining Catholic, several challenges arise regarding the three fundamental assumptions required for causal inference.

Causal Consistency: This requires that the outcome under each level of treatment to be compared is well-defined. In this context, defining what 'adopting Protestantism' and 'remaining Catholic' mean may present challenges. The practices and beliefs of each religion might vary significantly across countries and time periods, making it difficult to create a consistent, well-defined treatment. Furthermore, the outcome—economic development—may also be challenging to measure consistently across different countries and time periods.

There is undoubtedly considerable heterogeneity in the 'Protestant treatment.' In England, Protestantism was closely tied to the monarchy (Collinson, 2003). In Germany, Martin Luther's teachings emphasised individual faith in scripture, which, it has been claimed, supported economic development by promoting literacy (Gawthrop & Strauss, 1984). In England, King Henry VIII abolished Catholicism (Collinson, 2003). The Reformation, then, occurred differently in different places. The treatment needs to be better defined.

There is also ample scope for interference: 16th-century societies were interconnected through trade, diplomacy, and warfare. Thus, the religious decisions of one society were unlikely to have been independent from those of other societies.

Exchangeability: This requires that given the confounders, the potential outcomes are independent of the treatment assignment. It might be difficult to account for all possible confounders in this context. For example, historical, political, social, and geographical factors could influence both a country's religious affiliations and its economic development.

Positivity: This requires that there is a non-zero probability of every level of treatment for every stratum of confounders. If we consider various confounding factors such as geographical location, historical events, or political circumstances, some countries might only ever have the possibility of either remaining Catholic or becoming Protestant, but not both. For example, it is unclear under which conditions 16th-century Spain could have been randomly assigned to Protestantism (Nalle, 1987; Westreich & Cole, 2010).

Perhaps a more credible measure of effect in the region of our interests is the Average Treatment Effect in the Treated (ATT) expressed:

$$ATT_{\text{economic development}} = \mathbb{E}[(Y(a^*) - Y(a))|A = a^*, L]$$

Where $Y(a^*)$ represents the potential outcome if treated, and $Y(a)$ represents the potential outcome if not treated. The expectation is taken over the distribution of the treated units (i.e., those for whom $A = a^*$). L is a set of covariates on which we condition to ensure that the potential outcomes $Y(a^*)$ and $Y(a)$ are independent of the treatment assignment A, given L. This accounts for any confounding factors that might bias the estimate of the treatment effect.

Here, the ATT defines the expected difference in economic success for cultures that became Protestant compared with the expected economic success if those cultures had not become Protestant, conditional on measured confounders L, among the exposed ($A = a^*$). To estimate this contrast, our models would need to match Protestant cultures with comparable Catholic cultures effectively. By estimating the ATT, we avoid the assumption of non-deterministic positivity for the untreated. However, whether matching is conceptually plausible remains debatable. Ostensibly, it would seem that assigning a religion to a culture is not as easy as administering a pill (Watts et al., 2018).

S3. Causal Consistency Under Multiple Versions of Treatment

To better understand how the causal consistency assumption might fail, consider a question discussed in the evolutionary human science literature about whether a society's beliefs in big Gods affect its development of social complexity (Beheim et al., 2021; Johnson, 2015; Norenzayan et al., 2016; Sheehan et al., 2022; Slingerland et al., 2020; Watts et al., 2015; Whitehouse et al., 2023). Historians and anthropologists report that such beliefs vary over time and across cultures in intensity, interpretations, institutional management, and rituals (Bulbulia, J. et al., 2013; De Coulanges, 1903; Geertz et al., 2013; Wheatley, 1971). This variation in content and settings could influence social complexity. Moreover, the treatments realised in one society might affect those realised in other societies, resulting in *spill-over* effects in the exposures ('treatments') to be compared (Murray et al., 2021; Shiba et al., 2023).

The theory of causal inference under multiple versions of treatment, developed by VanderWeele and Hernán, formally addresses this challenge of treatment-effect heterogeneity (VanderWeele, 2009, 2018; VanderWeele & Hernan, 2013). The authors proved that if the treatment variations, K, are conditionally independent of the potential outcomes, $Y(k)$, given covariates L, then conditioning on L allows us to consistently estimate causal effects over the heterogeneous treatments (VanderWeele, 2009).

Where $\coprod$ denotes independence, we may assume causal consistency where the interventions to be compared are independent of their potential outcomes, conditional on covariates, L:

$$K \coprod Y(k)|L$$

According to the theory of causal inference under multiple versions of treatment, we may think of K as a 'coarsened indicator' for A. Although the theory of causal inference under multiple versions of treatment provides a formal solution to the problems of treatment-effect heterogeneity and treatment-effect dependencies (also known as SUTVA—the 'stable unit treatment value assumption'; refer to Rubin (1980)), computing and interpreting causal effect estimates under this theory can be challenging.

Consider the question of whether a reduction in Body Mass Index (BMI) affects health (Hernán & Taubman, 2008). Weight loss can occur through various methods, each with different health implications. Specific methods, such as regular exercise or a calorie-reduced diet, benefit health. However, weight loss might result from adverse conditions such as infectious diseases, cancers, depression, famine, or accidental amputations, which are generally not beneficial to health. Hence, even if causal effects of 'weight loss' could be consistently estimated when adjusting for covariates L, we might be uncertain about how to interpret the effect we are consistently estimating. This uncertainty highlights the need for precise and well-defined causal questions. For example, rather than stating the intervention vaguely as 'weight loss', we could state the intervention clearly and specifically as 'weight loss achieved through aerobic exercise over at least five years, compared with no weight loss.' This specificity in the definition of the treatment, along with comparable specificity in the statement of the outcomes, helps ensure that the causal estimates we obtain are not merely unbiased but also interpretable; for discussion, see Hernán et al. (2022); Murray et al. (2021); Hernán & Taubman (2008).

Beyond uncertainties for the interpretation of heterogeneous treatment effect estimates, there is the additional consideration that we cannot fully verify from data whether the measured covariates L suffice to render the multiple versions of treatment independent of the counterfactual outcomes. This problem is acute when there is *interference*, which occurs when treatment effects are relative to the density and distribution of treatment effects in a population. Scope for interference will often make it difficult to warrant the assumption that the potential outcomes are independent of the many versions of treatment that have been realised, dependently, on the administration of previous versions of treatments across the population (Bulbulia et al., 2023; Ogburn et al., 2022; VanderWeele & Hernan, 2013).

In short, although the theory of causal inference under multiple versions of treatment provides a formal solution for consistent causal effect estimation in observational settings, *treatment heterogeneity* remains a practical threat.

Generally, we should assume that causal consistency is unrealistic unless proven innocent.

For now, we note that the causal consistency assumption provides a theoretical starting point for recovering the missing counterfactuals required for computing causal contrasts. It identifies half of these missing counterfactuals directly from observed data. The concept of conditional exchangeability, which we examine next, offers a means for recovering the remaining half.

References

Basten, C., & Betz, F. (2013). Beyond work ethic: Religion, individual, and political preferences. *American Economic Journal: Economic Policy*, *5*(3), 67–91. https://doi.org/10.1257/pol.5.3.67

Becker, S. O., Pfaff, S., & Rubin, J. (2016). Causes and consequences of the protestant reformation. *Explorations in Economic History*, *62*, 1–25.

Beheim, B., Atkinson, Q. D., Bulbulia, J., Gervais, W., Gray, R. D., Henrich, J., Lang, M., Monroe, M. W., Muthukrishna, M., Norenzayan, A., Purzycki, B. G., Shariff, A., Slingerland, E., Spicer, R., & Willard, A. K. (2021). Treatment of missing data determined conclusions regarding moralizing gods. *Nature*, *595*(7866), E29–E34. https://doi.org/10.1038/s41586-021-03655-4

Bulbulia, J. A., Afzali, M. U., Yogeeswaran, K., & Sibley, C. G. (2023). Long-term causal effects of far-right terrorism in New Zealand. *PNAS Nexus*, *2*(8), pgad242.

Bulbulia, J., Geertz, A. W., Atkinson, Q. D., Cohen, E., Evans, N., Francois, P., Gintis, H., Gray, R. D., Henrich, J., Jordon, F. M., Norenzayan, A., Richerson, P. J., Slingerland, E., Turchin, P., Whitehouse, H., Widlok, T., & Wilson, D. S. (2013). *The cultural evolution of religion* (P. J. Richerson & M. Christiansen, Eds.; pp. 381–404). MIT press.

Collinson, P. (2003). *The reformation: A history*. Weidenfeld; Nicholson; London, England.

De Coulanges, F. (1903). *La cité antique: Étude sur le culte, le droit, les institutions de la grèce et de rome*. Hachette.

Gawthrop, R., & Strauss, G. (1984). Protestantism and literacy in early modern germany. *Past & Present*, *104*, 31–55.

Geertz, A. W., Atkinson, Q. D., Cohen, E., Evans, N., Francois, P., Gintis, H., Gray, R. D., Henrich, J., Jordon, F. M., Norenzayan, A., Richerson, P. J., Slingerland, E., Turchin, P., Whitehouse, H., Widlok, T., & Wilson, D. S. (2013). *The cultural evolution of religion* (P. J. Richerson & M. Christiansen, Eds.; pp. 381–404). MIT press.

Hernán, M. A., & Taubman, S. L. (2008). Does obesity shorten life? The importance of well-defined interventions to answer causal questions. *International Journal of Obesity (2005)*, *32 Suppl 3*, S8–14. https://doi.org/10.1038/ijo.2008.82

Hernán, M. A., Wang, W., & Leaf, D. E. (2022). Target trial emulation: A framework for causal inference from observational data. *JAMA*, *328*(24), 2446–2447. https://doi.org/10.1001/jama.2022.21383

Johnson, D. D. (2015). Big gods, small wonder: Supernatural punishment strikes back. *Religion, Brain & Behavior*, *5*(4), 290–298.

Murray, E. J., Marshall, B. D. L., & Buchanan, A. L. (2021). Emulating target trials to improve causal inference from agent-based models. *American Journal of Epidemiology*, *190*(8), 1652–1658. https://doi.org/10.1093/aje/kwab040

Nalle, S. T. (1987). Inquisitors, priests, and the people during the catholic reformation in spain. *The Sixteenth Century Journal*, 557–587.

Norenzayan, A., Shariff, A. F., Gervais, W. M., Willard, A. K., McNamara, R. A., Slingerland, E., & Henrich, J. (2016). The cultural evolution of prosocial religions. *Behavioral and Brain Sciences*, *39*, e1. https://doi.org/10.1017/S0140525X14001356

Ogburn, E. L., Sofrygin, O., Díaz, I., & Laan, M. J. van der. (2022). Causal inference for social network data. *Journal of the American Statistical Association*, *0*(0), 1–15. https://doi.org/10.1080/01621459.2022.2131557

Rubin, D. B. (1980). Randomization analysis of experimental data: The fisher randomization test comment. *Journal of the American Statistical Association*, *75*(371), 591–593. https://doi.org/10.2307/2287653

Sheehan, O., Watts, J., Gray, R. D., Bulbulia, J., Claessens, S., Ringen, E. J., & Atkinson, Q. D. (2022). Coevolution of religious and political authority in austronesian societies. *Nature Human Behaviour*. https://doi.org/10.1038/s41562-022-01471-y

Shiba, K., Daoud, A., Hikichi, H., Yazawa, A., Aida, J., Kondo, K., & Kawachi, I. (2023). Uncovering heterogeneous associations between disaster-related trauma and subsequent functional limitations: A machine-learning approach. *American Journal of Epidemiology*, *192*(2), 217–229.

Slingerland, E., Atkinson, Q. D., Ember, C. R., Sheehan, O., Muthukrishna, M., Bulbulia, J., & Gray, R. D. (2020). Coding culture: Challenges and recommendations for comparative cultural databases. *Evolutionary Human Sciences*, *2*, e29.

Swanson, G. E. (1967). *Religion and regime: A sociological account of the Reformation.*

Swanson, G. E. (1971). Interpreting the reformation. *The Journal of Interdisciplinary History*, *1*(3), 419–446. http://www.jstor.org/stable/202620

VanderWeele, T. J. (2009). Concerning the consistency assumption in causal inference. *Epidemiology*, *20*(6), 880. https://doi.org/10.1097/EDE.0b013e3181bd5638

VanderWeele, T. J. (2018). On well-defined hypothetical interventions in the potential outcomes framework. *Epidemiology*, *29*(4), e24. https://doi.org/10.1097/EDE.0000000000000823

VanderWeele, T. J., & Hernan, M. A. (2013). Causal inference under multiple versions of treatment. *Journal of Causal Inference*, *1*(1), 1–20.

Watts, J., Greenhill, S. J., Atkinson, Q. D., Currie, T. E., Bulbulia, J., & Gray, R. D. (2015). Broad supernatural punishment but not moralizing high gods precede the evolution of political complexity in Austronesia. In *Proceedings of the Royal Society B: Biological Sciences* (Vol. 282, p. 20142556). The Royal Society.

Watts, J., Sheehan, O., Bulbulia, Joseph A, Gray, R. D., & Atkinson, Q. D. (2018). Christianity spread faster in small, politically structured societies. *Nature Human Behaviour*, *2*(8), 559–564. https://doi.org/gdvnjn

Weber, M. (1905). *The protestant ethic and the spirit of capitalism: And other writings.* Penguin.

Weber, M. (1993). *The sociology of religion.* Beacon Press.

Westreich, D., & Cole, S. R. (2010). Invited commentary: positivity in practice. *American Journal of Epidemiology*, *171*(6). https://doi.org/10.1093/aje/kwp436

Wheatley, P. (1971). *The pivot of the four quarters : A preliminary enquiry into the origins and character of the ancient chinese city.* Edinburgh University Press. https://cir.nii.ac.jp/crid/1130000795717727104

Whitehouse, H., Francois, P., Savage, P. E., Hoyer, D., Feeney, K. C., Cioni, E., Purcell, R., Larson, J., Baines, J., Haar, B. ter, Covey, A., & Turchin, P. (2023). Testing the big gods hypothesis with global historical data: A review and retake. *Religion, Brain & Behavior*, *13*(2), 124–166.
